# Malaria-Infected Female Collared Flycatchers (*Ficedula albicollis*) Do Not Pay the Cost of Late Breeding

**DOI:** 10.1371/journal.pone.0085822

**Published:** 2014-01-23

**Authors:** Katarzyna Kulma, Matthew Low, Staffan Bensch, Anna Qvarnström

**Affiliations:** 1 Department of Ecology and Genetics, Uppsala University, Uppsala, Sweden; 2 Department of Ecology, Swedish University of Agricultural Sciences, Uppsala, Sweden; 3 Department of Biology, Lund University, Lund, Sweden; Arizona State University, United States of America

## Abstract

Life-history theory predicts that the trade-off between parasite defense and other costly traits such as reproduction may be most evident when resources are scarce. The strength of selection that parasites inflict on their host may therefore vary across environmental conditions. Collared flycatchers (*Ficedula albicollis*) breeding on the Swedish island Öland experience a seasonal decline in their preferred food resource, which opens the possibility to test the strength of life-history trade-offs across environmental conditions. We used nested-PCR and quantitative-PCR protocols to investigate the association of Haemosporidia infection with reproductive performance of collared flycatcher females in relation to a seasonal change in the external environment. We show that despite no difference in mean onset of breeding, infected females produced relatively more of their fledglings late in the season. This pattern was also upheld when considering only the most common malaria lineage (hPHSIB1), however there was no apparent link between the reproductive output and the intensity of infection. Infected females produced heavier-than-average fledglings with higher-than-expected recruitment success late in the season. This reversal of the typical seasonal trend in reproductive output compensated them for lower fledging and recruitment rates compared to uninfected birds earlier in the season. Thus, despite different seasonal patterns of reproductive performance the overall number of recruits was the same for infected versus uninfected birds. A possible explanation for our results is that infected females breed in a different microhabitat where food availability is higher late in the season but also is the risk of infection. Thus, our results suggest that another trade-off than the one we aimed to test is more important for explaining variation in reproductive performance in this natural population: female flycatchers appear to face a trade-off between the risk of infection and reproductive success late in the season.

## Introduction

Disease causing micro-parasites can have large negative impacts on the population dynamics of wild plants and animals [1]–[3], via pathogen virulence [4] and/or the costs of resistance [5], [6]. Since hosts are faced with trade-offs between their investments in immune function and other traits, the negative fitness effects of parasite infection may become more evident under stressful conditions such as low food abundance [4], [7], [8] or during periods of high investment in reproduction [9], [10]. This is because life-history theory predicts that the direction and magnitude of evolutionary trade-offs (such as between immune function and reproduction) will depend on the general availability of resources in the environment, together with individuals' ability to acquire and allocate these resources between traits [11], [12]. For example *Trypanosoma* prevalence markedly increased in Tengmalm's owls (*Aegolius funereus*) during years when their primary prey, *Microtus* voles, were at low levels [7] and wound healing in tree lizards (*Urosaurus ornatus*) was compromised when food resources were restricted [13]. These findings are consistent with the idea that trade-offs between immune function and reproduction should be most evident when resources are limited. Thus, understanding how hosts allocate resources between immunity and reproduction across various environmental contexts may be crucial for predicting how particular pathogens influence the host population dynamics and for increasing our general understanding of host-parasite co-evolution.

Haemosporidians (i.e. *Plasmodium*, *Haemoproteus* and *Leucocytozoon* genera.) are common blood parasites in birds and are found in virtually every avian family [14]. Owing to their wide geographical and host distribution, documented negative health effects (e.g. anemia, lethargy, [15]) and available molecular protocols for detection, Haemosporidia parasites have become an excellent model system for examining evolutionary and ecological aspects of host-parasite associations [9], [16]–[20]. Although experimental studies have been crucial in detecting the existence of fitness costs of blood parasitemia [21]–[23], there is still much uncertainty as to the magnitude and nature of these costs, and how they may change under different natural conditions.

Because of their high philopatry and breeding-site fidelity, collared flycatchers (*Ficedula albicollis*) provide an unusually good study system for investigating the fitness costs of blood parasites. On the Swedish island of Öland it is possible to accurately estimate not only reproductive performance of collared flycatchers, but also recruitment of offspring to the breeding population. Optimizing the date of egg laying so that the nestlings are brought up under optimal feeding conditions is crucial for maximizing reproductive success in birds [24]. Collared flycatchers experience a steep decline in reproductive success during the breeding season [25] that follows the seasonal decline in the abundance of their preferred food [26]. Therefore late breeding birds pay a fitness cost by failing to match their reproduction to the peak in primary prey (i.e. caterpillar) abundance. Because trade-offs between immune function and reproduction are expected to be greatest when resources are limited, we expect infected birds to show a steeper decline in reproductive performance as the breeding season progresses.

We used a nested PCR-protocol and sequencing to identify malaria infections and qPCR to measure infection intensity. We then investigated how infection with Haemosporidia parasites was linked with reproductive performance of female collared flycatchers in terms of clutch size, number of fledglings, fledgling condition and number of recruits relative to the timing of breeding. Our main aims were to answer the following questions: (i) do females infected with Haemosporidia parasites experience a steeper decline in reproductive success during the breeding season (i.e. when food resources become limited) as compared to uninfected females?; (ii) do infected females produce overall fewer fledglings and recruits and/or fledglings of lower quality?; and finally (iii) is there a higher reproductive cost of infection among birds with a higher intensity of infection?

## Materials and Methods

### Study system

Collared flycatchers colonized Öland, Sweden (57°10′N, 16°58′E) in the early 1960's, and their population has been intensively monitored since 2002 by regular inspection of over 2000 nest boxes in 21 woodlots across the island [27]. Males arrive at the breeding grounds in late April/early May and compete for natural nesting sites or nest boxes. Females usually arrive approximately a week after the males and select a breeding territory based on male and territory characteristics. Females usually lay only one clutch of 5–8 eggs per breeding season that they incubate for 13–16 days, with nestlings receiving biparental care until they fledge two weeks after hatching. Detailed records of breeding performance were recorded (see below) and all breeding birds were caught in the nest box in the middle of the egg-incubation period, then aged and sexed according to their plumage traits [28], ringed (if not banded before), measured, weighed (to nearest 0.1 g) and a small blood sample was taken for DNA and blood parasite analysis. Samples were collected on Öland between 2002 and 2010, except 2008 (for sample sizes see Table 1). Since the temporal pattern of food availability differs between deciduous and mixed forests, [26], we restricted our analysis to females breeding only in a deciduous (preferred) type of forest.

**Table 1:** Collared flycatcher females included in the study and their prevalence to Haemosporidia parasites.

Year	Females		Total N	Prevalence
	Infected	Uninfected		
**2002**	6	6	12	0.50
**2003**	21	16	37	0.57
**2004**	31	58	89	0.35
**2005**	29	45	74	0.39
**2006**	3	10	13	0.23
**2007**	15	16	31	0.48
**2009**	48	25	73	0.66
**2010**	15	12	27	0.56
**Total**	168	188	356	

### Ethics statement

This study was approved by the Swedish Board of Agriculture (*Jordbruksverket*; Permit numbers: M95-01, M 46-04 and Dnr 27-07).

### Recording reproductive variables

Nest boxes were inspected every third day during egg-laying to ensure accurate estimation of laying date and clutch size. Nestlings were measured, bled and individually ringed 7 days after hatching, and weighed and measured at 13 days of age. We estimated the number of fledglings as the number or nestlings present at 13 days, minus any dead fledglings found in the nest box post-fledging. Recruits are defined as offspring returning to the study area to breed in the subsequent years. We only consider female reproductive performance in this study, to avoid confounding effects in male fitness such as extra-pair copulations. Also, we excluded nests that were involved in any experimental manipulation [25], [29], [30]. These nests were randomly assigned to the experiments and they comprised only 16% of all clutches; therefore it is unlikely that their exclusion from the analysis affected our results.

### Avian malaria diagnosis

To classify infection status of breeding females we analyzed 356 blood samples (Table 1), 43 of which were from birds sampled more than once in the course of the study. Blood was collected from the brachial vein of each bird and stored in 99% ethanol (at room temperature) or in SET buffer (refrigerated) until extraction. DNA was extracted using a standard Proteinase K/SDS essay [31]. To assess the quality of DNA, 4 µL of each extract was run on 1.5% agarose gel stained in GelGreen. The concentration was quantified by spectrophotometer (Biophotometer, Eppendorf). The samples were screened for malaria using a PCR assay that amplifies a partial segment of the cytochrome *b* (cyt *b*) gene in the parasite mitochondria, using the same primers and reagents as in [32]. Negative control samples (with deionized water, dH_2_O, instead of genomic DNA), as well as positive control samples (*Haemoproteus pallidus* DNA) were routinely used: one negative control for every 11 samples, one positive control for every 40 samples. To avoid contamination, different sections of the laboratory were used for pre- and post-PCR processing. To investigate blood parasite prevalence, 3 µL of PCR product was visualized on 2% agarose gels stained with GelGreen. Samples showing a 525 bp-sized band were interpreted as a presence of parasitic DNA and were then prepared for sequencing using FastAP™ (Fermentas). Purified DNA fragments were sent to Macrogen Inc., Seoul, Korea, for sequencing. The resulting sequences were aligned and compared with the MalAvi database [33] using the BioEdit© software. Information about morphospecies of respective malaria lineages was obtained from the MalAvi database [33].

### Infection intensity analysis – quantitative PCR protocol

We quantified *Haemoproteus* infection in 112 samples (out of 133, 84% of all *Haemoproteus*-infected samples). Extracted DNA was diluted to a concentration of approximately 1 ng/µl, which later served as template DNA. We carried out the real-time quantitative PCR for quantification of *Haemoproteus* infections, using genus-specific primers that amplify a portion of the cyt *b* gene without co-amplification of *Plasmodium* parasites (Supplementary Table S4). Also, to get an accurate estimation of host DNA concentration, a second reaction was performed that amplifies a single copy of conserved nuclear sequence in host DNA region [34]. Both protocols were almost identical regarding the reaction composition and thermal cycle profile and differed only in annealing temperature. Thus, each reaction of 25 µl contained 5 µl of DNA, 12.5 µl Platinum SYBR Green qPCR SuperMix-UDG (Life Technologies™), 10 pmol of each primer, 0.1 µl ROX dye and 5.4 µl ddH_2_O. The thermal reaction profile was as follows: after the initial incubation at 50°C for 2 mins, 42 cycle were run in 95°C for 15 secs, then in 56°C for *Haemoproteus* primers (and 57°C for host primers) for 30 secs and 72°C for 30 secs. All qPCR reactions were performed in Mx3000P real time PCR instrument (Stratagene). Each sample was run in duplicates and its value was calculated from the mean. In parasitemia quantification, after a preliminary analysis, we chose the sample with the highest parasitemia (hPHSIB1 lineage, *Haemoproteus majoris* morphospecies) and made a series of five steps −5× dilutions. We run these −5× dilution series as a standard along with our samples to estimate the relative parasitemia. For host DNA quantification, we produced standard curves by making five step −5× dilutions (using ddH_2_O) of flycatcher DNA, starting from the highest concentration of 5 ng/µl. The efficiency of qPCR reactions was high, ranging from 98.8–102.2% (standard curves with slopes between −3.35 and −3.27). The individual dissociation curves were inspected to verify specific amplification. Finally, we adjusted the relative parasitemia by the total content of host DNA in each reaction.

### Statistical analysis

We used linear mixed (LMM) and generalized linear mixed models (GLMM) with Poisson error distribution to investigate the association between female infection status (i.e. infected with Haemosporidia or not) and the following estimates of reproductive performance; timing of breeding, clutch size, number of fledged offspring, number of recruited offspring, fledgling condition (average fledgling weight), and number of recruited offspring. Using the same methods we also investigated the association between infection intensity (i.e. *Haemoproteus*-infection intensity among the infected birds) and the same estimates of reproductive performance as listed above. Since the mean population breeding date differed between years due to changes in weather conditions, we used year-standardized lay date residuals (residuals from lay date by year ANOVA) in our statistical models. Age was included as a fixed factor in addition to infection status because older birds are known to breed earlier and to produce more fledglings and recruits [35]. Year and bird identity were included as random factors in all models and when investigating the link between female infection status and fledgling condition we also included nest identity to control for a common rearing environment. The data for fledgling weight was box-cox transformed (and divided by 1000 to keep the parameter values low; (fledgling weight)^4/5^/1000.) to meet the parametric assumptions. All Poisson models were checked for overdispersion by extracting the Pearson residuals and when needed, individual identity was included as a random effect to account for overdispersion.

To account for model uncertainty in model selection we used candidate model sets that included all combinations of the main effects and their interactions. The relative strength of support for each model was determined using AIC with a second-order correction for sample size (AICc) and the AICc model weight [36]. All models within 2 AICc of the highest-ranked model (i.e. ΔAICc<2) were model-averaged to calculate predictor estimates and standard errors using full-model averaging method [36], [37]. Also, based on these models we calculated the cumulative evidence (importance weights) and 95% confidence intervals for each predictor in order to identify factors with the highest predictive value. We highlight the model parameters where confidence intervals do not overlap with zero to show high support for the direction of an effect. All statistical analyses described here were conducted using R version 2.15.0 [38], with packages lme4 (model generation, (g)lmer function [39]) and MuMIn (model selection and model averaging [40]).

## Results

### General seasonal pattern of reproductive performance

In agreement with previous findings, the reproductive performance of female collared flycatchers breeding in our study sites showed a significant decline with progressing breeding season in terms of clutch size (z_1,1849_ = −4.89, p<0.0001) and number of fledglings (z_1,1624_ = −8.82, p<0.0001) produced. Thus, later breeding females generally laid fewer eggs and produced fewer fledglings. Moreover, nestlings hatched late in the season had a lower chance of recruiting into the population in the following years (z_1,1624_ = −6.55, p<0.0001).

### General pattern of *Haemosporidia* infection and lineage-specific prevalence

The prevalence of malaria infection in the collared flycatcher population was 47%, but there was significant variation between the eight study years (GLZ with binomial errors and logit link function, z_1,354_ = −2.888, p = 0.004, see Table 1). One-year-old birds were less often infected than older birds (z_1,354_ = 3.573, p<0.001, the proportion of yearlings was 0.34). Overall, there was no significant difference in the timing of breeding between infected and uninfected flycatcher females (t_1,344_ = 1.14, p>0.1). The most common lineage among infected females was hPHSIB1 (*Haemoproteus majoris* morphospecies) that accounted for 68% of all infections for which we had information about the lineage identity (Supplementary Table S1). As the second most common lineage (hCOLL2; *Haemoproteus pallidus* morphospecies) accounted for only 12% of the infections (Supplementary Table S1), we restricted our lineage-specific analysis of the association between infection and reproductive performance to birds infected with the most common malaria strain (hPHSIB1).

### Infection status and seasonal variation in reproductive performance

#### Clutch size

**Figure 1:**
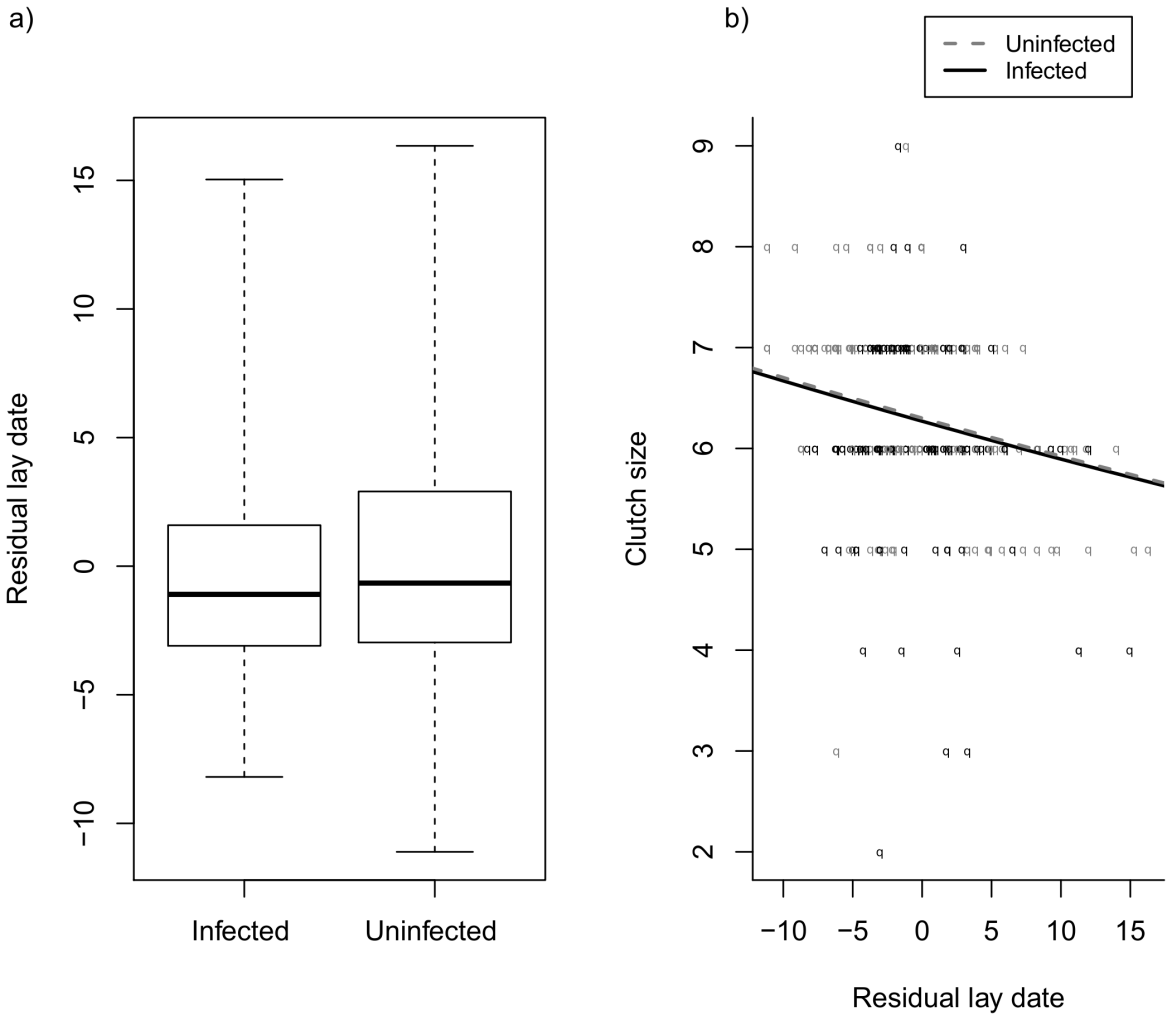
Comparison of onset of breeding and clutch size in relation to breeding time between female collared flycatchers infected and uninfected with Haemosporidia parasites breeding on Swedish island of Öland. In onset of breeding graph a), thick horizontal segments represent median, the bottom and the upper edges of the boxes show first and third quartiles, whiskers demonstrate minimum and maximum values. In clutch size graph b), the slopes represent predicted values derived from model-averaged estimates based on models within ΔAICc<2. Infected and uninfected females do not differ in their onset of breeding or in their seasonal decline in clutch size.

Female collared flycatchers produced smaller clutch sizes as the breeding season progressed but we found no evidence for a steeper decline in clutch size among the infected females (Table 2, Fig. 1).

**Table 2:** Summary of GLMM reproductive success models for collared flycatcher females.

Factor	Averaged parameter estimate ± SE	Lower 95% CI	Upper 95% CI	Relative importance weight
**(a) Lay date**				
Age (yearlings)	1.20±0.62	−0.004	2.408	1.00
Infection (Uninfected)	0.46±0.56	−0.636	1.546	0.55
Age*Infection	−0.33±1.06	−2.405	1.748	0.27
**(b) Clutch size**				
Residual lay date	−0.008±0.005	−0.018	0.001	0.76
Age (Yearlings)	−0.034±0.046	−0.124	0.055	0.19
Infection (Uninfected)	0.028±0.043	−0.056	0.112	0.17
**(c) Number of fledglings**				
Residual lay date	−0.007±0.012	−0.031	0.016	1.00
Infection (Uninfected)	0.03±0.05	−0.078	0.130	0.60
Lay date*Infection	−0.02±0.01	−0.050	0.000	0.47
Age (Yearlings)	0.05±0.05	−0.053	0.159	0.32
**(d) Number of recruits**				
Residual lay date	−0.005±0.039	−0.071	0.082	1.00
Infection (Uninfected)	−0.05±0.19	−0.316	0.416	1.00
Lay date*Infection	**−0.14±0.05**	**−0.229**	**−0.049**	**1.00**
Age (Yearlings)	0.17±0.18	−0.187	0.519	0.35
**(e) Number of fledglings per laid egg**				
Age (Yearlings)	0.14±0.08	−0.028	0.305	0.74
Infection (Uninfected)	0.07±0.07	−0.064	0.203	0.22
Age * Infection	−0.18±0.11	−0.409	0.046	0.22
**(f) Number of recruit to fledglings ratio**				
Residual lay date	−0.005±0.041	−0.075	0.085	1.00
Infection (Uninfected)	0.04±0.19	−0.323	0.410	1.00
Lay date*Infection	**−0.12±0.05**	**−0.218**	**−0.030**	**1.00**
Age (Yearlings)	0.15±0.18	−0.203	0.495	0.33
**(g) Average fledgling weight**				
**Infection (Uninfected)**	**−0.35±0.14**	**−0.614**	**−0.081**	**1.00**

Averaged parameter estimates and importance weights were derived from the models for which ΔAIC_c_<2. Bold font points at variables for which model estimates are different from 0.

#### Number of fledglings and recruits

**Figure 2:**
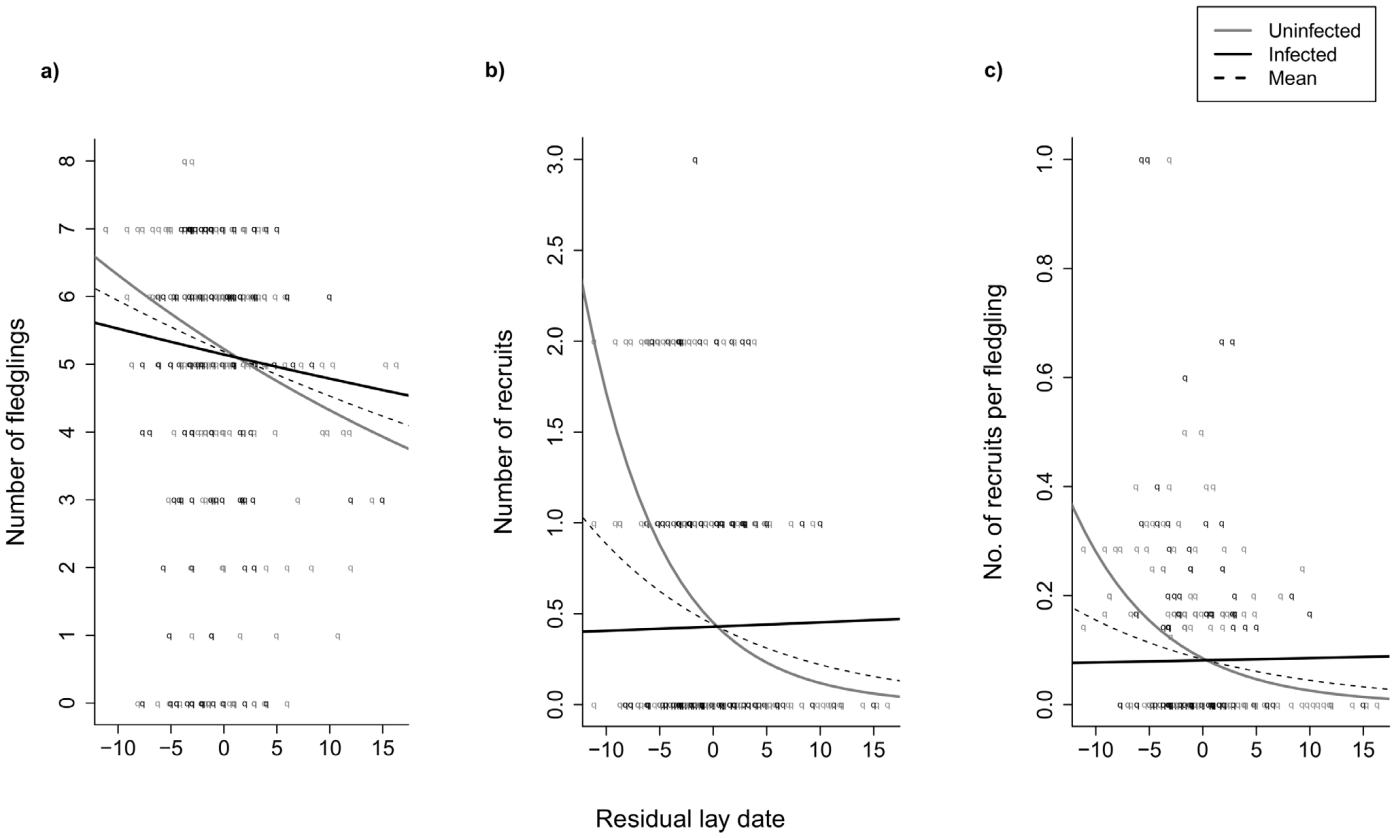
Comparison of the seasonal pattern of reproductive performance between female collared flycatchers infected with blood parasites (solid black line), uninfected females (solid grey line) and the mean for all analyzed females (dashed black line) breeding on the Swedish island of Öland. It was plotted with model-averaged (ΔAICc<2) prediction lines using shrinkage estimates: a) number of fledglings, b) number of recruits, c) number of recruits per fledged offspring. Uninfected females show a typical seasonal decline in reproductive success where they rear more fledglings and recruits early in the season, whereas females infected with Haemosporidia parasites show an opposite pattern. As a result, uninfected females rear proportionally more recruits per fledgling early in the season. Infected females, however, experience a much shallower decline in recruits as the breeding season progresses, and thus recruit the same number of offspring. Despite altered pattern among infected females, the trend for the population remains negative.

Infected and uninfected females showed different seasonal patterns of reproductive performance in terms of the number of fledglings produced. Uninfected females experienced the typical seasonal decline in number of fledglings (slope lower 95%CI = −0.037, upper 95%CI = −0.009), whereas females with haemosporidian infections did not (Table 2; Fig. 2, the slope was not significantly different from zero: slope lower 95%CI = −0.019, upper 95%CI = 0.021). Infected females produced fewer fledglings when compared to uninfected females early in the season, but relatively more fledglings late in the season. This model with interaction between residual lay date and infection status had the lowest AIC value among candidate models; the interaction alone showed a moderate, but significant support (Supplementary Table S2c, but see Table 2). Because infected and uninfected females differed in the seasonal pattern of fledgling- but not egg-production, we investigated if those two groups of females also differed in proportion of reared fledged offspring per laid egg: it was done using logged values of clutch size as an offset variable with Poisson error structure. As judged from our models, (Table 2, Supplementary Table S2f) there was no such difference.

Because post-fledging survival generally declines with later breeding dates, uninfected females maximized fledgling production when offspring are more likely to survive in the consecutive year (i.e. early in the season). We therefore expected infected females to produce fewer recruits compared to uninfected females. However, there was no apparent difference in number of recruits between infected and uninfected females (GLMM with Poisson error distribution and year and bird identity as random factors; Tables 2&3). To investigate whether infected females simply produced more fledglings to account for achieving the same number of recruits, or had higher survival prospects per number of fledglings produced, we tested whether the two groups of females differed in their ratio of the recruits per fledged offspring (rec/fledge ratio). Such an estimate gives an indication of the relative reproductive investment and parental effort put into producing the same number of recruits. There was no overall difference in the ratio of recruits per fledged offspring between infected and uninfected females (Table 3), because infected females produced more recruits per fledged offspring later in the season (Table 2, Fig. 2c). Thus, contrary to expectations, infected females produced the same number of fledglings as uninfected females, with a similar survival probability despite being reared later in the season, i.e. when resources are expected to be scarce.

**Table 3:** Mixed Model (Residual lay date) and Generalized Linear Mixed Model estimates with 95% confidence intervals (breeding year and bird identity as random factors).

Response variable	Model Estimate	Model estimate SE	Lower 95% CI	Upper 95% CI	Statistic value	N
Residual lay date	0.584	0.493	−0.382	1.550	t = 1.19	346
Clutch size	0.024	0.043	−0.060	0.108	z = 0.55	346
Number of fledglings	0.031	0.051	−0.069	0.131	z = 0.61	302
Number of recruits	0.241	0.171	−0.094	0.576	z = 1.41	302
Fledglings to clutch size ratio	0.015	0.051	−0.085	0.115	z = 0.29	308
Recruits to fledglings ratio	0.235	0.171	−0.100	0.570	z = 1.38	276

Infected and uninfected females do not differ in reproductive output (model estimates are not different from 0). Infected females served as a reference group.

### Infection status and fledgling condition

**Figure 3:**
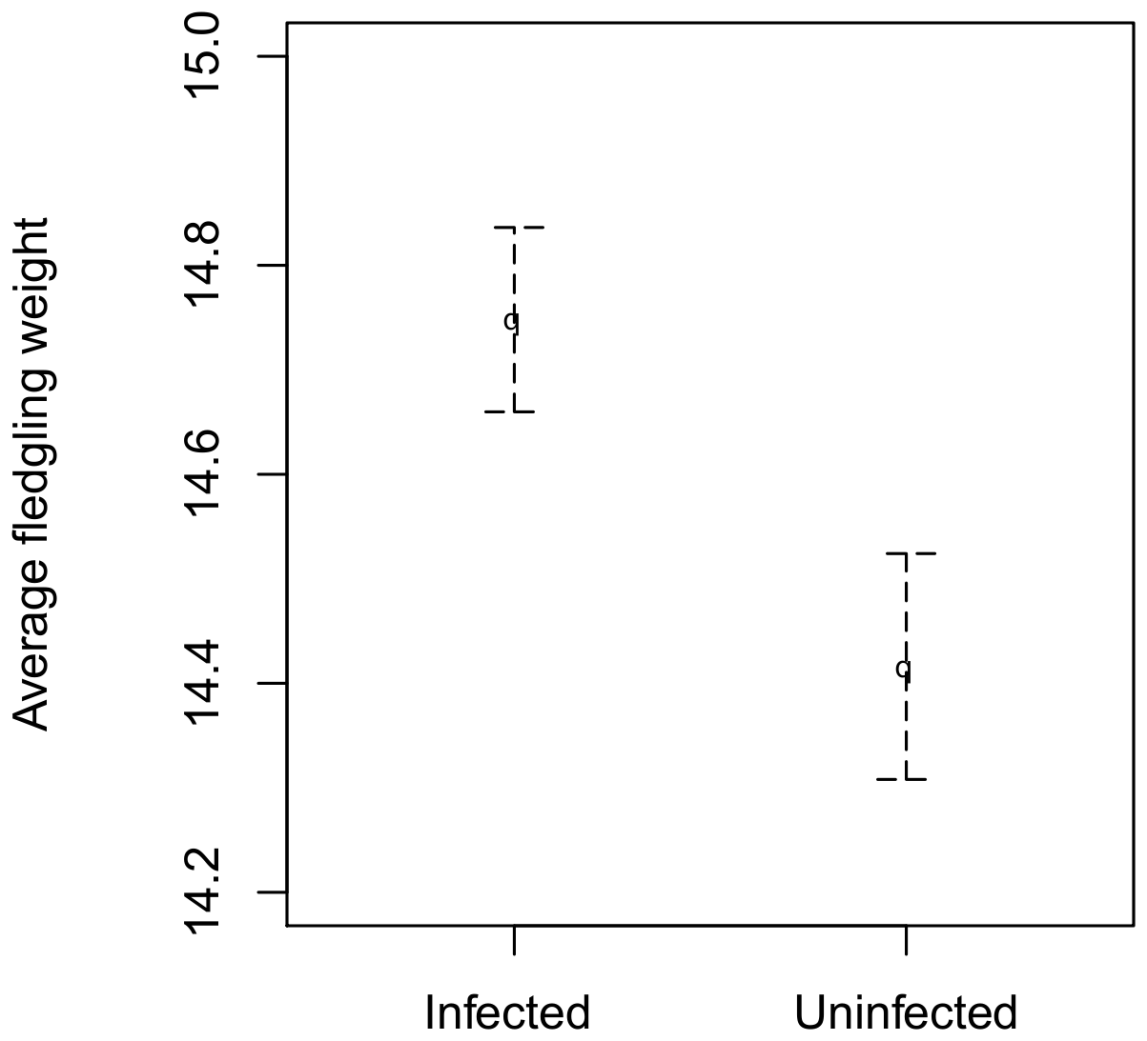
Comparison of fledgling weight of Haemosporidia-infected and uninfected collared flycatcher females. Fledglings of infected females are on average heavier than offspring of uninfected females. The plot dots show means for respective groups of females, error bars indicate 1 standard error from the mean.

Because infected females produced fledglings with good survival prospects, despite them being mostly reared late in the season, we investigated whether there were any differences in average body weight when 13 days old: a factor known to influence fledgling survival. Interestingly, infected females reared heavier fledglings than uninfected females (Table 2) but there was no support for an interaction between infection status and residual lay date (Table 2; Fig. 3).

### Intensity of *Haemoproteus* infections in relation to reproductive output and fledgling condition

We performed similar analyses of reproductive success and fledgling quality in relation to infection intensity of females infected with *Haemoproteus* parasites. Parasitemia did not differ across the breeding season (GLMM, t_1,128_ = −0.54, p>0.1). Moreover, none of the models supported a role of intensity of infection in any aspect of reproductive performance, in most cases including a null model within ΔAIC_c_<2 (Supplementary Table S5).

### *Haemoproteus majoris* (hPHSIB1) infections in relation to reproductive output and fledgling condition

We repeated our analysis of reproductive success and fledgling quality with regard to females infected only with the most common malaria lineage (hPHSIB1) compared to those free from infections. These results essentially mirrored our previous findings, with infected females showing a different seasonal pattern of reproductive success to uninfected females: i.e. a relatively higher production of fledged and recruited offspring later in the season and rearing on average heavier fledglings (Table 4, Supplementary Table S3). However, we did not find substantial differences in average fledging weight between the offspring reared by hPHSIB1-infected and uninfected females (Table 4).

**Table 4:** Summary of GLMM reproductive success models for collared flycatcher females either infected with hPHSIB1 lineage or uninfected.

Factor	Averaged parameter estimate ± SE	Lower 95% CI	Upper 95% CI	Relative importance weight
**(a) Lay date**				
Age (yearlings)	0.99±0.76	−0.489	2.475	0.83
Infection (Uninfected)	0.16±0.67	−1.159	1.479	0.45
Age*Infection	−0.02±1.28	−2.536	2.498	0.24
**(b) Clutch size**				
Residual lay date	−0.009±0.005	−0.019	0.000	0.82
Age (Yearlings)	−0.05±0.05	−0.144	0.049	0.23
Infection (Uninfected)	0.03±0.05	−0.061	0.131	0.19
**(c) Number of fledglings**				
Residual lay date	−0.007**±**0.013	−0.033	0.018	1.00
Infection (Uninfected)	0.06±0.05	−0.056	0.180	0.73
Lay date*Infection	−0.025±0.013	−0.052	0.002	0.51
Age (Yearlings)	−0.035±0.059	−0.080	0.150	0.15
**(d) Number of recruits**				
Residual lay date	−0.014±0.045	−0.073	0.101	1.00
Infection (Uninfected)	−0.06±0.21	−0.364	0.477	1.00
Lay date*Infection	**−0.15**±**0.05**	**−0.247**	**−0.046**	**1.00**
Age (Yearlings)	0.13±0.19	−0.248	0.515	0.30
**(e) Fledglings to clutch size ratio**				
Age (Yearlings)	0.08±0.06	−0.043	0.197	0.36
Infection (Uninfected)	0.02±0.06	−0.105	0.144	0.17
**(f) Recruits to fledglings ratio**				
Residual lay date	−0.01±0.06	−0.131	0.105	1.00
Infection (Uninfected)	−0.002±0.215	−0.419	0.423	0.79
Lay date*Infection	**−0.13±0.05**	**−0.233**	**−0.022**	**0.79**
Age	0.12±0.19	−0.255	0.502	0.24
**(g) Fledgling weight**				
Infection (Uninfected)	−0.30±0.17	−0.63	0.02	0.41

Averaged parameter estimates and importance weights were derived from the models for which ΔAIC_c_<2. Bold font points at variables for which model estimates are different from 0.

## Discussion

Female collared flycatchers infected by Haemosporidia parasites began breeding at a similar time as uninfected females, but showed a different seasonal pattern of reproductive performance: early in the season (i.e. when resources are expected to be most abundant) infected females produced fewer fledglings than uninfected individuals, with this pattern reversing late in the season (Fig. 2). We found similar results when we narrowed the analysis to the most common blood parasite lineage (hPHSIB1). Interestingly, infected females reared heavier 13-day old nestlings than uninfected individuals. The estimated intensity of *Haemoproteus*-infection did not, however, explain any aspect of reproductive success in this flycatcher population.

Uninfected females showed a typical seasonal decline in reproductive performance (Fig. 2), which is thought to be related to seasonal changes in caterpillar availability within deciduous forest [26], [41]. In such habitats, caterpillar abundance shows an early and narrow peak [26] and early-reared nestlings have more time to develop before the autumn migration. Consequently, early breeders generally produce twice as many recruits compared to late breeding pairs [42], although the temporal pattern of both food abundance and reproductive success can differ between years due to weather conditions [43]. As chronic malaria infections are expected to have negative effects on multiple breeding stages, e.g. hatching success, provisioning rate and fledgling success [21]–[23], we expected that infected females should experience a steeper decline in reproductive performance as compared to uninfected females. In contrast to this expectation we found that infected females did not show a seasonal decline in reproductive performance measured in terms of number of recruited offspring. In fact, late in the season, during potentially more demanding conditions, infected females had even higher breeding success than uninfected females. There are at least two possible explanations to these findings: first, parasitized females may perform a terminal investment in current reproduction; and/or second, they may breed in microhabitats with a different seasonal food distribution.

According to life-history theory, infected females may invest more in current breeding if they are less likely to survive and reproduce in the future, performing a so-called terminal investment [35], [44]. Late-breeding females may compensate for poorer prospects of future breeding by increased investment in reproduction and parental care. Early in the season, when fledged offspring have a higher likelihood of survival and food resources are abundant, all females should make significant investments in current reproduction. In contrast, late in the season, when the prospect of producing recruits is much lower, uninfected individuals may reduce their current investment in reproduction (and start preparing for their own migration) while infected females with lower survival prospects may continue to invest in current reproduction. If so, the pattern early in the season would reflect variation in resource acquisition among individuals (i.e. uninfected individuals do best because they are in better shape and can bring more food) and the pattern late in the season would reflect variation in allocation (infected individuals do best because they allocate more resources to current reproduction and work harder). However, infected females do not have a lower survival than their uninfected counterparts in our population [45], so they do not have lower prospects of future breeding, which rejects the core assumption of the terminal investment hypothesis.

Another possible explanation for this pattern is that infected females may breed in a different microhabitat where the food distribution is more stable across the season, but where also the risk of infection is higher. Usually the forests with more coniferous trees show a different temporal distribution of food with a lower, slightly later and more stable caterpillar abundance throughout the season [26], but we excluded females breeding in mixed forests from our analysis. However, the microhabitat varies within study plots (e.g. by type of water source in the area and temperature), suggesting that both food availability and vector abundance - and thus risk of infection - are linked to these microhabitat variables [46]. It is also possible that the vectors themselves serve as an alternative food resource that is more evenly distributed across the breeding season.

Studies of resident blue tits have found striking differences in spatial and temporal distribution of malaria infections over as short distance as one kilometer [47], [48]. Although this structure results from a complex interaction between multiple biogeographical factors (e.g. local landscape features, host state, parasite genotype, etc.), the distance from a big body of water (The River Thames) well predicted the risk of infection [47]. Interestingly, preliminary analyses performed on collared flycatchers from Öland indicate a link between the risk of malaria infection and the distance from the water source (Kulma et al., unpublished data). However, we need more detailed epidemiological studies to better understand factors driving the unexpected pattern of reproductive performance of infected birds in our population.

The major challenge for studies on life-history evolution in natural populations is not to establish the existence of particular trade-offs, but rather which of their possible combinations occur and are strongest [49]. We did not find any support for a major role of the trade-off we initially aimed to investigate (i.e. between infection status and reproductive performance) in explaining variation in the temporal pattern of reproductive performance in collared flycatchers. Instead, our results suggest that late breeding birds face a trade-off between minimizing the risk of infection and reproductive performance.

### Conclusions

We found that infected and uninfected female collared flycatchers did not differ in their mean onset of breeding but they nevertheless showed a different seasonal pattern of reproductive performance. In contrast to our prediction, infected females did not show a steeper seasonal decline in reproductive performance as compared to uninfected females. Instead, infected females produced a larger proportion of their fledglings late in the season when post-fledging survival is usually lower, but they did not produce fewer fledglings or recruits compared to uninfected females. Moreover, their fledglings were on average heavier than fledglings of uninfected mothers, despite being reared later in the breeding season. Thus, female collared flycatchers with haemosporidian infections show a non-typical seasonal pattern of reproductive performance, suggesting that they breed in territories where the seasonal food distribution is different and not declining across the season.

## Supporting Information

Table S1 **Diversity of avian malaria lineages in collared flycatcher females from Öland.** Prevalence was obtained by dividing a number of specific lineage infections by the number of all identified infections. The lineages marked as “Unknown” are probably transmitted in Africa, as they have been found in tropical migrants in Europe but only in adults.
(DOC)

Table S2 **Reproductive success model selection tables for all individuals.**
(DOC)

Table S3 **Reproductive success model selection tables for individuals infected with hPHSIB1 lineage or uninfected females.**
(DOC)

Table S4 **Primer sequences used for real-time qPCR.** Genus-specific primers amplify a fragment of parasite's cytochrome b gene; host primers amplify an ultra-conserved of the host nuclear DNA.
(DOC)

Table S5 **Reproductive success and infection intensity model selection tables for a subset (see** **Materials and Methods** **section for details) individuals infected with hPHSIB1 lineage or uninfected females.**
(DOC)
