# Supplementary material for: Malaria-Infected Female Collared Flycatchers (Ficedula albicollis) Do Not Pay the Cost of Late Breeding
Source: PLoS One. 2014 Jan 23;9(1):e85822. doi: 10.1371/journal.pone.0085822 (PMC3900437; doi:10.1371/journal.pone.0085822)
Supplement: Table S1 — Diversity of avian malaria lineages in collared flycatcher females from Öland. Prevalence was obtained by dividing a number of specific lineage infections by the number of all identified infections. The lineages marked as “Unknown” are probably transmitted in Africa, as they have been found in tropical migrants in Europe but only in adults. (DOC) [file pone.0085822.s001.doc]

| **Parasite Taxon** | **Lineage** | **Area of Transmission** | **Fraction of identified infections % (N)** |
| --- | --- | --- | --- |
|
| *Haemoproteus pallidus* | hCOLL2 | Africa | 12.1 (19) |
| *Haemoproteus balmorali* | hCOLL3 | Unknown | 2.0 (3) |
| *Haemoproteus pallidus* | hPFC1 | Europe | 2.5 (4) |
| *Haemoproteus majoris* | hPHSIB1 | Europe | 68.1 (107) |
| *Plasmodium sp.* | pACCTAC01 | Africa | 0.6 (1) |
| *Plasmodium sp.* | pCOLL4 | Unknown | 1.3 (2) |
| *Plasmodium sp.* | pCOLL7 | Africa | 2.0 (3) |
| *Plasmodium sp.* | pCOLL10 | Unknown | 0.6 (1) |
| *Plasmodium sp.* | pCOLL11 | Unknown | 0.6 (1) |
| *Plasmodium sp.* | pGRW07 | Unknown | 0.6 (1) |
| *Plasmodium sp.* | pGRW09 | Africa | 1.3 (2) |
| *Plasmodium sp.* | pGRW11 | Europe | 0.6 (1) |
| *Plasmodium sp.* | pLAMPUR03 | Africa | 1.3 (2) |
| *Plasmodium sp.* | pPBPIP1 | Africa | 0.6 (1) |
| *Plasmodium sp.* | pRTSR1 | Africa | 1.3 (2) |
| *Plasmodium relictum* | pSGS1 | Europe & Africa | 0.6 (1) |
| *Plasmodium sp.* | pSYBOR10 | Africa | 1.3 (2) |
| *Plasmodium sp.* | pTERUF02 | Africa | 0.6 (1) |
| *Plasmodium circumflexum* | pTURDUS1 | Europe | 0.6 (1) |
| *Plasmodium sp.* | pWW4 | Unknown | 1.3 (2) |
|  |  |  |  |
|  | **Total identified** |  | 157 |
|  | **Total unidentified** |  | 11 |
|  |  |  |  |
|  | **Total infected** |  | 168 |
|  | **Total uninfected** |  | 188 |
|  | **Total screened** |  | 356 |

**Appendix S1.** Diversity of avian malaria lineages in collared flycatcher females from Öland. Prevalence was obtained by dividing a number of specific lineage infections by the number of all identified infections. The lineages marked as “Unknown” are probably transmitted in Africa, as they have been found in tropical migrants in Europe but only in adults.
