# Supplementary material for: Malaria-Infected Female Collared Flycatchers (Ficedula albicollis) Do Not Pay the Cost of Late Breeding
Source: PLoS One. 2014 Jan 23;9(1):e85822. doi: 10.1371/journal.pone.0085822 (PMC3900437; doi:10.1371/journal.pone.0085822)
Supplement: Table S2 — Reproductive success model selection tables for all individuals. (DOC) [file pone.0085822.s002.doc]

**Appendix S2**. Reproductive success model selection tables

1. Lay date

i) All lay date models

| Model | Age | Infection | Age * Infection | k | Log Likelihood | AICc | Δ AICc | Weight |
| --- | --- | --- | --- | --- | --- | --- | --- | --- |
| 2 | + |  |  | 5 | -1012.795 | 2035.8 | 0.00 | 0.401 |
| 4 | + | + |  | 6 | -1012.250 | 2036.7 | 0.98 | 0.246 |
| 8 | + | + | + | 7 | -1011.226 | 2036.8 | 1.02 | 0.241 |
| 1 |  |  |  | 4 | -1015.733 | 2039.6 | 3.82 | 0.059 |
|  |  | + |  | 5 | -1014.821 | 2039.8 | 4.05 | 0.053 |

ii) Lay date models with Δ AICc < 2

| Model | Age | Infection | Age * Infection | k | Log Likelihood | AICc | Δ AICc | Weight |
| --- | --- | --- | --- | --- | --- | --- | --- | --- |
| 2 | + |  |  | 5 | -1012.795 | 2035.8 | 0.00 | 0.45 |
| 4 | + | + |  | 6 | -1012.250 | 2036.7 | 0.98 | 0.28 |
| 8 | + | + | + | 7 | -1011.226 | 2036.8 | 1.02 | 0.27 |

iii) Lay date: relative variable importance based on a-ii)

| Age | Infection | Age * Infection |
| --- | --- | --- |
| 1.00 | 0.55 | 0.27 |

1. Clutch size

i) All clutch size models

| Model | Residual Lay date | Age | Infection | Lay date* Age | Lay Date* Infection | Age* Infection | Lay date* Age* Infection | k | Log Likelihood | AICc | Δ AICc | Weight |
| --- | --- | --- | --- | --- | --- | --- | --- | --- | --- | --- | --- | --- |
| 2 | + |  |  |  |  |  |  | 4 | -21.349 | 50.8 | 0.00 | 0.231 |
| 1 |  |  |  |  |  |  |  | 3 | -22.855 | 51.8 | 0.97 | 0.143 |
| 4 | + | + |  |  |  |  |  | 5 | -21.062 | 52.3 | 1.49 | 0.110 |
| 6 | + |  | + |  |  |  |  | 5 | -21.137 | 52.4 | 1.63 | 0.102 |
| 3 |  | + |  |  |  |  |  | 4 | -22.389 | 52.9 | 2.08 | 0.082 |
| 5 |  |  | + |  |  |  |  | 4 | -22.703 | 53.5 | 2.71 | 0.060 |
| 8 | + | + | + |  |  |  |  | 6 | -20.745 | 53.7 | 2.92 | 0.054 |
| 22 | + |  | + |  | + |  |  | 6 | -21.032 | 54.3 | 3.50 | 0.040 |
| 12 | + | + |  | + |  |  |  | 6 | -21.059 | 54.4 | 3.55 | 0.039 |
| 7 |  | + | + |  |  |  |  | 5 | -22.117 | 54.4 | 3.59 | 0.038 |
| 24 | + | + | + |  | + |  |  | 7 | -20.628 | 55.6 | 4.77 | 0.021 |
| 40 | + | + | + |  |  | + |  | 7 | -20.694 | 55.7 | 4.90 | 0.020 |
| 16 | + | + | + | + |  |  |  | 7 | -20.740 | 55.8 | 5.00 | 0.019 |
| 39 |  | + | + |  |  | + |  | 6 | -22.072 | 56.4 | 5.58 | 0.014 |
| 56 | + | + | + |  | + | + |  | 8 | -20.594 | 57.6 | 6.80 | 0.008 |
| 32 | + | + | + | + | + |  |  | 8 | -20.619 | 57.7 | 6.85 | 0.008 |
| 48 | + | + | + | + |  | + |  | 8 | -20.689 | 57.8 | 6.99 | 0.007 |
| 64 | + | + | + | + | + | + |  | 9 | -20.586 | 59.7 | 8.89 | 0.003 |
| 128 | + | + | + | + | + | + | + | 10 | -20.469 | 61.6 | 10.78 | 0.001 |

ii) Clutch size models with Δ AICc < 2

| Model | Residual Lay date | Age | Infection | Lay date* Age | Lay Date* Infection | Age* Infection | Lay date* Age* Infection | k | Log Likelihood | AICc | Δ AICc | Weight |
| --- | --- | --- | --- | --- | --- | --- | --- | --- | --- | --- | --- | --- |
| 2 | + |  |  |  |  |  |  | 4 | -21.349 | 50.8 | 0.00 | 0.39 |
| 1 |  |  |  |  |  |  |  | 3 | -22.855 | 51.8 | 0.97 | 0.24 |
| 4 | + | + |  |  |  |  |  | 5 | -21.062 | 52.3 | 1.49 | 0.19 |
| 6 | + |  | + |  |  |  |  | 5 | -21.137 | 52.4 | 1.63 | 0.17 |

iii) Clutch size: relative variable importance based on b-ii)

| Residual Lay Date | Age | Infection |
| --- | --- | --- |
| 0.76 | 0.19 | 0.17 |

1. Number of fledglings

i) All number of fledglings models

| Model | Residual Lay date | Age | Infection | Lay date* Age | Lay Date* Infection | Age* Infection | Lay date* Age* Infection | k | Log Likelihood | AICc | Δ AICc | Weight |
| --- | --- | --- | --- | --- | --- | --- | --- | --- | --- | --- | --- | --- |
| 22 | + |  | + |  | + |  |  | 6 | -95.144 | 202.6 | 0.00 | 0.188 |
| 2 | + |  |  |  |  |  |  | 4 | -97.449 | 203.0 | 0.46 | 0.149 |
| 24 | + | + | + |  | + |  |  | 7 | -94.730 | 203.8 | 1.27 | 0.100 |
| 4 | + | + |  |  |  |  |  | 5 | -96.882 | 204.0 | 1.40 | 0.094 |
| 6 | + |  | + |  |  |  |  | 5 | -97.067 | 204.3 | 1.76 | 0.078 |
| 12 | + | + |  | + |  |  |  | 6 | -96.189 | 204.7 | 2.09 | 0.066 |
| 32 | + | + | + | + | + |  |  | 8 | -94.107 | 204.7 | 2.13 | 0.065 |
| 56 | + | + | + |  | + | + |  | 8 | -94.314 | 205.1 | 2.55 | 0.053 |
| 8 | + | + | + |  |  |  |  | 6 | -96.628 | 205.5 | 2.97 | 0.043 |
| 64 | + | + | + | + | + | + |  | 9 | -93.699 | 206.0 | 3.44 | 0.034 |
| 40 | + | + | + |  |  | + |  | 7 | -95.915 | 206.2 | 3.64 | 0.030 |
| 16 | + | + | + | + |  |  |  | 7 | -95.933 | 206.2 | 3.68 | 0.030 |
| 48 | + | + | + | + |  | + |  | 8 | -95.290 | 207.1 | 4.50 | 0.020 |
| 1 |  |  |  |  |  |  |  | 3 | -100.613 | 207.3 | 4.73 | 0.018 |
| 128 | + | + | + | + | + | + | + | 10 | -93.582 | 207.9 | 5.35 | 0.013 |
| 3 |  | + |  |  |  |  |  | 4 | -100.427 | 209.0 | 6.42 | 0.008 |
| 5 |  |  | + |  |  |  |  | 4 | -100.429 | 209.0 | 6.42 | 0.008 |
| 7 |  | + | + |  |  |  |  | 5 | -100.298 | 210.8 | 8.23 | 0.003 |
| 39 |  | + | + |  |  | + |  | 6 | -99.493 | 211.3 | 8.70 | 0.002 |

ii) Number of fledgling models with Δ AICc < 2

| Model | Residual Lay date | Age | Infection | Lay date* Age | Lay Date* Infection | Age* Infection | Lay date* Age* Infection | k | Log Likelihood | AICc | Δ AICc | Weight |
| --- | --- | --- | --- | --- | --- | --- | --- | --- | --- | --- | --- | --- |
| 22 | + |  | + |  | + |  |  | 6 | -95.144 | 202.6 | 0.00 | 0.31 |
| 2 | + |  |  |  |  |  |  | 4 | -97.449 | 203.0 | 0.46 | 0.25 |
| 24 | + | + | + |  | + |  |  | 7 | -94.730 | 203.8 | 1.27 | 0.16 |
| 4 | + | + |  |  |  |  |  | 5 | -96.882 | 204.0 | 1.40 | 0.15 |
| 6 | + |  | + |  |  |  |  | 5 | -97.067 | 204.3 | 1.76 | 0.13 |
|  |  |  |  |  |  |  |  |  |  |  |  |  |

iii) Number of fledglings: relative variable importance based on c-ii)

| Residual Lay Date | Infection | Lay Date* Infection | Age |
| --- | --- | --- | --- |
| 1.00 | 0.60 | 0.47 | 0.32 |

1. Number of recruits

i) All number of recruits models

| Model | Residual Lay date | Age | Infection | Lay date* Age | Lay Date* Infection | Age* Infection | Lay date* Age* Infection | k | Log Likelihood | AICc | Δ AICc | Weight |
| --- | --- | --- | --- | --- | --- | --- | --- | --- | --- | --- | --- | --- |
| 22 | + |  | + |  | + |  |  | 6 | -124.053 | 260.4 | 0.00 | 0.459 |
| 24 | + | + | + |  | + |  |  | 7 | -123.631 | 261.6 | 1.25 | 0.245 |
| 56 | + | + | + |  | + | + |  | 8 | -123.474 | 263.4 | 3.05 | 0.100 |
| 32 | + | + | + | + | + |  |  | 8 | -123.612 | 263.7 | 3.32 | 0.087 |
| 64 | + | + | + | + | + | + |  | 9 | -123.428 | 265.5 | 5.08 | 0.036 |
| 6 | + |  | + |  |  |  |  | 5 | -128.379 | 267.0 | 6.57 | 0.017 |
| 128 | + | + | + | + | + | + | + | 10 | -123.342 | 267.4 | 7.05 | 0.014 |
| 2 | + |  |  |  |  |  |  | 4 | -129.805 | 267.7 | 7.35 | 0.012 |
| 8 | + | + | + |  |  |  |  | 6 | -127.957 | 268.2 | 7.81 | 0.009 |
| 4 | + | + |  |  |  |  |  | 5 | -129.175 | 268.6 | 8.16 | 0.008 |
| 40 | + | + | + |  |  | + |  | 7 | -127.440 | 269.3 | 8.87 | 0.005 |
| 16 | + | + | + | + |  |  |  | 7 | -127.941 | 270.3 | 9.87 | 0.003 |
| 12 | + | + |  | + |  |  |  | 6 | -129.171 | 270.6 | 10.24 | 0.003 |
| 48 | + | + | + | + |  | + |  | 8 | -127.424 | 271.3 | 10.95 | 0.002 |
| 1 |  |  |  |  |  |  |  | 3 | -140.617 | 287.3 | 26.92 | 0.000 |
| 5 |  |  | + |  |  |  |  | 4 | -139.604 | 287.3 | 26.95 | 0.000 |
| 3 |  | + |  |  |  |  |  | 4 | -140.575 | 289.3 | 28.89 | 0.000 |
| 7 |  | + | + |  |  |  |  | 5 | -139.600 | 289.4 | 29.01 | 0.000 |
| 39 |  | + | + |  |  | + |  | 6 | -138.783 | 289.9 | 29.46 | 0.000 |

ii) Number of recruit models with Δ AICc < 2

| Model | Residual Lay date | Age | Infection | Lay date* Age | Lay Date* Infection | Age* Infection | Lay date* Age* Infection | k | Log Likelihood | AICc | Δ AICc | Weight |
| --- | --- | --- | --- | --- | --- | --- | --- | --- | --- | --- | --- | --- |
| 22 | + |  | + |  | + |  |  | 6 | -124.053 | 260.4 | 0.00 | 0.65 |
| 24 | + | + | + |  | + |  |  | 7 | -123.631 | 261.6 | 1.25 | 0.35 |
|  |  |  |  |  |  |  |  |  |  |  |  |  |

iii) Recruit relative variable importance based on d-ii)

| Residual Lay Date | Infection | Lay Date* Infection | Age |
| --- | --- | --- | --- |
| 1.00 | 1.00 | 1.00 | 0.35 |

1. Number of recruit to fledglings ratio
2. All recruit to fledglings ratio models

| Model | Residual Lay date | Age | Infection | Lay date* Age | Lay Date* Infection | Age* Infection | Lay date* Age* Infection | k | Log Likelihood | AICc | Δ AICc | Weight |
| --- | --- | --- | --- | --- | --- | --- | --- | --- | --- | --- | --- | --- |
| 22 | + |  | + |  | + |  |  | 6 | -118.102 | 248.5 | 0.00 | 0.423 |
| 24 | + | + | + |  | + |  |  | 7 | -117.767 | 249.9 | 1.43 | 0.207 |
| 56 | + | + | + |  | + | + |  | 8 | -117.686 | 251.9 | 3.38 | 0.078 |
| 32 | + | + | + | + | + |  |  | 8 | -117.767 | 252.0 | 3.54 | 0.072 |
| 2 | + |  |  |  |  |  |  | 4 | -122.324 | 252.8 | 4.29 | 0.049 |
| 6 | + |  | + |  |  |  |  | 5 | -121.298 | 252.8 | 4.31 | 0.049 |
| 64 | + | + | + | + | + | + |  | 9 | -117.686 | 254.0 | 5.50 | 0.027 |
| 4 | + | + |  |  |  |  |  | 5 | -121.897 | 254.0 | 5.51 | 0.027 |
| 8 | + | + | + |  |  |  |  | 6 | -121.004 | 254.3 | 5.81 | 0.023 |
| 128 | + | + | + | + | + | + | + | 10 | -117.442 | 255.6 | 7.15 | 0.012 |
| 40 | + | + | + |  |  | + |  | 7 | -120.727 | 255.8 | 7.35 | 0.011 |
| 12 | + | + |  | + |  |  |  | 6 | -121.867 | 256.0 | 7.53 | 0.010 |
| 16 | + | + | + | + |  |  |  | 7 | -120.995 | 256.4 | 7.88 | 0.008 |
| 48 | + | + | + | + |  | + |  | 8 | -120.719 | 257.9 | 9.44 | 0.004 |
| 1 |  |  |  |  |  |  |  | 3 | -130.518 | 267.1 | 18.63 | 0.000 |
| 5 |  |  | + |  |  |  |  | 4 | -129.555 | 267.2 | 18.76 | 0.000 |
| 3 |  | + |  |  |  |  |  | 4 | -130.469 | 269.1 | 20.58 | 0.000 |
| 7 |  | + | + |  |  |  |  | 5 | -129.545 | 269.3 | 20.80 | 0.000 |
| 39 |  | + | + |  |  | + |  | 6 | -128.990 | 270.3 | 21.78 | 0.000 |

ii) Recruits to fledglings ratio models with Δ AICc < 2

| Model | Residual Lay date | Age | Infection | Lay date* Age | Lay Date* Infection | Age* Infection | Lay date* Age* Infection | k | Log Likelihood | AICc | Δ AICc | Weight |
| --- | --- | --- | --- | --- | --- | --- | --- | --- | --- | --- | --- | --- |
| 22 | + |  | + |  | + |  |  | 6 | -118.102 | 248.5 | 0.00 | 0.67 |
| 24 | + | + | + |  | + |  |  | 7 | -117.767 | 249.9 | 1.43 | 0.33 |

1. Number of recruits to fledglings ratio: relative variable importance based on g-ii)

| Residual Lay Date | Infection | Infection* Lay Date | Age |
| --- | --- | --- | --- |
| 1.00 | 1.00 | 1.00 | 0.33 |

1. Number of fledglings to clutch size ratio
2. All fledglings to clutch size ratio models

| Model | Residual Lay date | Age | Infection | Lay date* Age | Lay Date* Infection | Age* Infection | Lay date* Age* Infection | k | Log Likelihood | AICc | Δ AICc | Weight |
| --- | --- | --- | --- | --- | --- | --- | --- | --- | --- | --- | --- | --- |
| 3 |  | + |  |  |  |  |  | 4 | -211.071 | 430.3 | 0.00 | 0.219 |
| 1 |  |  |  |  |  |  |  | 3 | -212.785 | 431.6 | 1.37 | 0.110 |
| 39 |  | + | + |  |  | + |  | 6 | -209.842 | 432.0 | 1.69 | 0.094 |
| 4 | + | + |  |  |  |  |  | 5 | -211.057 | 432.3 | 2.04 | 0.079 |
| 7 |  | + | + |  |  |  |  | 5 | -211.060 | 432.3 | 2.05 | 0.079 |
| 24 | + | + | + |  | + |  |  | 7 | -209.355 | 433.1 | 2.81 | 0.054 |
| 12 | + | + |  | + |  |  |  | 6 | -210.601 | 433.5 | 3.21 | 0.044 |
| 5 |  |  | + |  |  | + |  | 4 | -212.687 | 433.5 | 3.23 | 0.043 |
| 56 | + | + | + |  | + |  |  | 8 | -208.533 | 433.6 | 3.28 | 0.042 |
| 2 | + |  |  |  |  |  |  | 4 | -212.772 | 433.7 | 3.40 | 0.040 |
| 40 | + | + | + |  |  | + |  | 7 | -209.835 | 434.1 | 3.77 | 0.033 |
| 22 | + |  | + |  | + |  |  | 6 | -210.891 | 434.1 | 3.79 | 0.033 |
| 8 | + | + | + |  |  |  |  | 6 | -211.044 | 434.4 | 4.10 | 0.028 |
| 32 | + | + | + | + | + |  |  | 8 | -208.989 | 434.5 | 4.19 | 0.027 |
| 64 | + | + | + | + | + | + |  | 9 | -208.183 | 435.0 | 4.71 | 0.021 |
| 48 | + | + | + | + |  | + |  | 8 | -209.460 | 435.4 | 5.13 | 0.017 |
| 16 | + | + | + | + |  |  |  | 7 | -210.585 | 435.6 | 5.27 | 0.016 |
| 6 | + |  | + |  |  |  |  | 5 | -212.680 | 435.6 | 5.29 | 0.016 |
| 128 | + | + | + | + | + | + | + | 10 | -208.146 | 437.0 | 6.77 | 0.007 |

ii) Fledglings to clutch size ratio with Δ AICc < 2

| Model | Residual Lay date | Age | Infection | Lay date* Age | Lay Date* Infection | Age* Infection | Lay date* Age* Infection | k | Log Likelihood | AICc | Δ AICc | Weight |
| --- | --- | --- | --- | --- | --- | --- | --- | --- | --- | --- | --- | --- |
| 3 |  | + |  |  |  |  |  | 4 | -211.071 | 430.3 | 0.00 | 0.52 |
| 1 |  |  |  |  |  |  |  | 3 | -212.785 | 431.6 | 1.37 | 0.26 |
| 39 |  | + | + |  |  | + |  | 6 | -209.842 | 432.0 | 1.69 | 0.22 |

1. Fledglings to clutch size ratio: relative variable importance based on g-ii)

| Age | Infection | Age*Infection |
| --- | --- | --- |
| 0.74 | 0.22 | 0.22 |

1. Average fledgling weight models

i) All average fledgling weight models

| Model | Residual Lay date | Age | Infection | Lay date* Age | Lay Date* Infection | Age* Infection | Lay date* Age* Infection | k | Log Likelihood | AICc | Δ AICc | Weight |
| --- | --- | --- | --- | --- | --- | --- | --- | --- | --- | --- | --- | --- |
| 5 |  |  | + |  |  |  |  | 5 | -207.614 | 425.6 | 0.00 | 0.620 |
| 1 |  |  |  |  |  |  |  | 4 | -209.683 | 427.6 | 2.01 | 0.227 |
| 7 |  | + | + |  |  |  |  | 6 | -208.581 | 429.7 | 4.09 | 0.080 |
| 3 |  | + |  |  |  |  |  | 5 | -210.576 | 431.5 | 5.92 | 0.032 |
| 39 |  | + | + |  |  | + |  | 7 | -208.827 | 432.4 | 6.77 | 0.021 |
| 6 | + |  | + |  |  |  |  | 6 | -210.669 | 433.9 | 8.27 | 0.010 |
| 2 | + |  |  |  |  |  |  | 5 | -212.677 | 435.7 | 10.13 | 0.004 |
| 16 | + | + | + | + | + |  |  | 8 | -210.015 | 437.0 | 11.36 | 0.002 |
| 8 | + | + | + |  |  |  |  | 7 | -211.555 | 437.8 | 12.22 | 0.001 |
| 4 | + | + |  |  |  |  |  | 6 | -213.461 | 439.5 | 13.85 | 0.001 |
| 48 | + | + | + | + |  | + |  | 9 | -210.158 | 439.5 | 13.89 | 0.001 |
| 12 | + | + |  | + |  |  |  | 7 | -212.672 | 440.1 | 14.46 | 0.000 |
| 22 | + |  | + |  | + |  |  | 7 | -212.698 | 440.1 | 14.51 | 0.000 |
| 40 | + | + | + |  |  | + |  | 8 | -211.822 | 440.6 | 14.97 | 0.000 |
| 32 | + | + | + | + | + |  |  | 9 | -212.162 | 443.5 | 17.90 | 0.000 |
| 24 | + |  | + |  | + |  |  | 8 | -213.588 | 444.1 | 18.51 | 0.000 |
| 64 | + |  | + | + | + | + |  | 10 | -212.144 | 445.7 | 20.13 | 0.000 |
| 128 | + |  | + | + | + | + | + | 11 | -211.168 | 446.1 | 20.48 | 0.000 |
| 56 | + |  | + |  | + | + |  | 9 | -213.737 | 446.7 | 21.05 | 0.000 |

ii) Average fledgling weight models with Δ AICc < 2

| Model | Residual Lay date | Age | Infection | Lay date* Age | Lay Date* Infection | Age* Infection | Lay date* Age* Infection | k | Log Likelihood | AICc | Δ AICc | Weight |
| --- | --- | --- | --- | --- | --- | --- | --- | --- | --- | --- | --- | --- |
| 5 |  |  | + |  |  |  |  | 5 | -207.614 | 425.6 | 0.00 | 1.00 |

iii) Average fledgling weight: relative variable importance based on g-ii)

| Infection |
| --- |
| 1.00 |
