# Supplementary material for: Malaria-Infected Female Collared Flycatchers (Ficedula albicollis) Do Not Pay the Cost of Late Breeding
Source: PLoS One. 2014 Jan 23;9(1):e85822. doi: 10.1371/journal.pone.0085822 (PMC3900437; doi:10.1371/journal.pone.0085822)
Supplement: Table S3 — Reproductive success model selection tables for individuals infected with hPHSIB1 lineage or uninfected females. (DOC) [file pone.0085822.s003.doc]

**Appendix S3**. Reproductive success model selection tables for individuals infected with hPHSIB1 lineage or uninfected females

1. Lay date

i) All lay date models

| Model | Age | Infection | Age * Infection | k | Log Likelihood | AICc | Δ AICc | Weight |
| --- | --- | --- | --- | --- | --- | --- | --- | --- |
| 2 | + |  |  | 5 | -858.753 | 1727.7 | 0.00 | 0.346 |
| 8 | + | + | + | 7 | -857.136 | 1728.7 | 0.95 | 0.215 |
| 4 | + | + |  | 6 | -858.304 | 1728.9 | 1.19 | 0.191 |
| 1 |  |  |  | 4 | -860.601 | 1729.3 | 1.63 | 0.154 |
| 3 |  | + |  | 5 | -860.061 | 1730.3 | 2.62 | 0.094 |

ii) Lay date models with Δ AICc < 2

| Model | Age | Infection | Age * Infection | k | Log Likelihood | AICc | Δ AICc | Weight |
| --- | --- | --- | --- | --- | --- | --- | --- | --- |
| 2 | + |  |  | 5 | -858.753 | 1727.7 | 0.00 | 0.38 |
| 8 | + | + | + | 7 | -857.136 | 1728.7 | 0.95 | 0.24 |
| 4 | + | + |  | 6 | -858.304 | 1728.9 | 1.19 | 0.21 |
| 1 |  |  |  | 4 | -860.601 | 1729.3 | 1.63 | 0.17 |

iii) Lay date: relative variable importance based on a-ii)

| Age | Infection | Age * Infection |
| --- | --- | --- |
| 0.83 | 0.45 | 0.24 |

1. Clutch size

i) All clutch size models

| Model | Residual Lay date | Age | Infection | Lay date* Age | Lay Date* Infection | Age* Infection | Lay date* Age* Infection | k | Log Likelihood | AICc | Δ AICc | Weight |
| --- | --- | --- | --- | --- | --- | --- | --- | --- | --- | --- | --- | --- |
| 2 | + |  |  |  |  |  |  | 4 | -16.558 | 41.3 | 0.00 | 0.232 |
| 4 | + | + |  |  |  |  |  | 5 | -16.089 | 42.4 | 1.13 | 0.132 |
| 6 | + |  | + |  |  |  |  | 5 | -16.304 | 42.8 | 1.56 | 0.106 |
| 1 |  |  |  |  |  |  |  | 3 | -18.391 | 42.9 | 1.61 | 0.104 |
| 3 |  | + |  |  |  |  |  | 4 | -17.725 | 43.6 | 2.33 | 0.072 |
| 8 | + | + | + |  |  |  |  | 6 | -15.700 | 43.7 | 2.44 | 0.069 |
| 12 | + | + |  |  |  |  |  | 6 | -16.074 | 44.4 | 3.19 | 0.047 |
| 5 |  |  | + |  |  |  |  | 4 | -18.163 | 44.5 | 3.21 | 0.047 |
| 22 | + |  | + |  | + |  |  | 6 | -16.295 | 44.9 | 3.63 | 0.038 |
| 7 |  | + | + |  |  |  |  | 5 | -17.342 | 44.9 | 3.64 | 0.038 |
| 16 | + | + | + | + |  |  |  | 7 | -15.687 | 45.8 | 4.52 | 0.024 |
| 24 | + | + | + |  | + |  |  | 7 | -15.692 | 45.8 | 4.53 | 0.024 |
| 40 | + | + | + |  |  | + |  | 7 | -15.696 | 45.8 | 4.53 | 0.024 |
| 39 |  | + | + |  |  | + |  | 6 | -17.339 | 47.0 | 5.72 | 0.013 |
| 32 | + | + | + | + | + |  |  | 8 | -15.677 | 47.9 | 6.61 | 0.009 |
| 48 | + | + | + | + |  | + |  | 8 | -15.682 | 47.9 | 6.62 | 0.008 |
| 56 | + | + | + |  | + | + |  | 8 | -15.686 | 47.9 | 6.63 | 0.008 |
| 64 | + | + | + | + | + | + |  | 9 | -15.671 | 50.0 | 8.73 | 0.003 |
| 128 | + | + | + | + | + | + | + | 10 | -15.570 | 51.9 | 10.68 | 0.001 |

ii) Clutch size models with Δ AICc < 2

| Model | Residual Lay date | Age | Infection | Lay date* Age | Lay Date* Infection | Age* Infection | Lay date* Age* Infection | k | Log Likelihood | AICc | Δ AICc | Weight |
| --- | --- | --- | --- | --- | --- | --- | --- | --- | --- | --- | --- | --- |
| 2 | + |  |  |  |  |  |  | 4 | -16.558 | 41.3 | 0.00 | 0.40 |
| 4 | + | + |  |  |  |  |  | 5 | -16.089 | 42.4 | 1.13 | 0.23 |
| 6 | + |  | + |  |  |  |  | 5 | -16.304 | 42.8 | 1.56 | 0.19 |
| 1 |  |  |  |  |  |  |  | 3 | -18.391 | 42.9 | 1.61 | 0.18 |

iii) Clutch size: relative variable importance based on b-ii)

| Residual Lay Date | Age | Infection |
| --- | --- | --- |
| 0.82 | 0.23 | 0.19 |

1. Number of fledglings

i) All number of fledglings models

| Model | Residual Lay date | Age | Infection | Lay date* Age | Lay Date* Infection | Age* Infection | Lay date* Age* Infection | k | Log Likelihood | AICc | Δ AICc | Weight |
| --- | --- | --- | --- | --- | --- | --- | --- | --- | --- | --- | --- | --- |
| 22 | + |  | + |  | + |  |  | 6 | -79.378 | 171.1 | 0.00 | 0.207 |
| 2 | + |  |  |  |  |  |  | 4 | -81.741 | 171.6 | 0.54 | 0.158 |
| 6 | + |  | + |  |  |  |  | 5 | -80.925 | 172.1 | 0.99 | 0.126 |
| 24 | + | + | + |  | + |  |  | 7 | -79.199 | 172.9 | 1.76 | 0.086 |
| 4 | + | + |  |  |  |  |  | 5 | -81.443 | 173.1 | 2.03 | 0.075 |
| 32 | + | + | + | + | + |  |  | 8 | -78.526 | 173.6 | 2.55 | 0.058 |
| 12 | + | + |  | + |  |  |  | 6 | -80.739 | 173.8 | 2.72 | 0.053 |
| 8 | + | + | + |  |  |  |  | 6 | -80.751 | 173.8 | 2.75 | 0.052 |
| 16 | + | + | + | + |  |  |  | 7 | -80.034 | 174.5 | 3.43 | 0.037 |
| 56 | + | + | + |  | + | + |  | 8 | -78.995 | 174.6 | 3.48 | 0.036 |
| 40 | + | + | + |  |  | + |  | 7 | -80.360 | 175.2 | 4.08 | 0.027 |
| 64 | + | + | + | + | + | + |  | 9 | -78.314 | 175.4 | 4.27 | 0.024 |
| 48 | + | + | + | + |  | + |  | 8 | -79.677 | 175.9 | 4.85 | 0.018 |
| 1 |  |  |  |  |  |  |  | 3 | -85.273 | 176.6 | 5.54 | 0.013 |
| 128 | + | + | + | + | + | + | + | 10 | 78.088 | 177.1 | 5.99 | 0.010 |
| 5 |  |  | + |  |  |  |  | 4 | 84.662 | 177.5 | 6.39 | 0.008 |
| 3 |  | + |  |  |  |  |  | 4 | -85.218 | 178.6 | 7.50 | 0.005 |
| 7 |  | + | + |  |  |  |  | 5 | -84.649 | 179.5 | 8.44 | 0.003 |
| 39 |  | + | + |  |  | + |  | 6 | -84.201 | 180.7 | 9.65 | 0.002 |

ii) Number of fledgling models with Δ AICc < 2

| Model | Residual Lay date | Age | Infection | Lay date* Age | Lay Date* Infection | Age* Infection | Lay date* Age* Infection | k | Log Likelihood | AICc | Δ AICc | Weight |
| --- | --- | --- | --- | --- | --- | --- | --- | --- | --- | --- | --- | --- |
| 22 | + |  | + |  | + |  |  | 6 | -79.378 | 171.1 | 0.00 | 0.36 |
| 2 | + |  |  |  |  |  |  | 4 | -81.741 | 171.6 | 0.54 | 0.27 |
| 6 | + |  | + |  |  |  |  | 5 | -80.925 | 172.1 | 0.99 | 0.22 |
| 24 | + | + | + |  | + |  |  | 7 | -79.199 | 172.9 | 1.76 | 0.15 |

iii) Number of fledglings: relative variable importance based on c-ii)

| Residual Lay Date | Infection | Lay Date* Infection | Age |
| --- | --- | --- | --- |
| 1.00 | 0.73 | 0.51 | 0.15 |

1. Number of recruits

i) All number of recruits models

| Model | Residual Lay date | Age | Infection | Lay date* Age | Lay Date* Infection | Age* Infection | Lay date* Age* Infection | k | Log Likelihood | AICc | Δ AICc | Weight |
| --- | --- | --- | --- | --- | --- | --- | --- | --- | --- | --- | --- | --- |
| 22 | + |  | + |  | + |  |  | 6 | -103.119 | 218.6 | 0.00 | 0.481 |
| 24 | + | + | + |  | + |  |  | 7 | -102.885 | 220.2 | 1.65 | 0.211 |
| 32 | + | + | + | + | + |  |  | 8 | -102.778 | 222.2 | 3.57 | 0.081 |
| 56 | + | + | + |  | + | + |  | 8 | -102.811 | 222.2 | 3.63 | 0.078 |
| 64 | + | + | + | + | + | + |  | 9 | -102.660 | 224.1 | 5.49 | 0.031 |
| 6 | + |  | + |  |  |  |  | 5 | -106.948 | 224.1 | 5.56 | 0.030 |
| 2 | + |  |  |  |  |  |  | 4 | -108.015 | 224.2 | 5.61 | 0.029 |
| 4 | + | + |  |  |  |  |  | 5 | -107.657 | 225.6 | 6.98 | 0.015 |
| 8 | + | + | + |  |  |  |  | 6 | -106.702 | 225.7 | 7.17 | 0.013 |
| 128 | + | + | + | + | + | + | + | 10 | -102.659 | 226.2 | 7.65 | 0.010 |
| 40 | + | + | + |  |  | + |  | 7 | -106.312 | 227.1 | 8.50 | 0.007 |
| 12 | + | + |  | + |  |  |  | 6 | -107.529 | 227.4 | 8.82 | 0.006 |
| 16 | + | + | + | + |  |  |  | 7 | -106.555 | 227.6 | 8.99 | 0.005 |
| 48 | + | + | + | + |  | + |  | 8 | -106.160 | 228.9 | 10.33 | 0.003 |
| 1 |  |  |  |  |  |  |  | 3 | -119.443 | 245.0 | 26.40 | 0.000 |
| 5 |  |  | + |  |  |  |  | 4 | -118.585 | 245.3 | 26.75 | 0.000 |
| 3 |  | + |  |  |  |  |  | 4 | -119.442 | 247.0 | 28.46 | 0.000 |
| 7 |  | + | + |  |  |  |  | 5 | -118.565 | 247.4 | 28.79 | 0.000 |
| 39 |  | + | + |  |  | + |  | 6 | -118.040 | 248.4 | 29.84 | 0.000 |

ii) Number of recruit models with Δ AICc < 2

| Model | Residual Lay date | Age | Infection | Lay date* Age | Lay Date* Infection | Age* Infection | Lay date* Age* Infection | k | Log Likelihood | AICc | Δ AICc | Weight |
| --- | --- | --- | --- | --- | --- | --- | --- | --- | --- | --- | --- | --- |
| 22 | + |  | + |  | + |  |  | 6 | -103.119 | 218.6 | 0.00 | 0.7 |
| 24 | + | + | + |  | + |  |  | 7 | -102.885 | 220.2 | 1.65 | 0.3 |

iii) Recruit relative variable importance based on d-ii)

| Residual Lay Date | Infection | Lay Date* Infection | Age |
| --- | --- | --- | --- |
| 1.0 | 1.0 | 1.0 | 0.3 |

1. Number of recruit to fledglings ratio
2. All recruit to fledglings ratio models

| Model | Residual Lay date | Age | Infection | Lay date* Age | Lay Date* Infection | Age* Infection | Lay date* Age* Infection | k | Log Likelihood | AICc | Δ AICc | Weight |
| --- | --- | --- | --- | --- | --- | --- | --- | --- | --- | --- | --- | --- |
| 22 | + |  | + |  | + |  |  | 6 | -96.420 | 205.2 | 0.00 | 0.358 |
| 24 | + | + | + |  | + |  |  | 7 | -96.216 | 206.9 | 1.71 | 0.152 |
| 2 | + |  |  |  |  |  |  | 4 | -99.471 | 207.1 | 1.92 | 0.137 |
| 6 | + |  | + |  |  |  |  | 5 | -99.043 | 208.3 | 3.15 | 0.074 |
| 4 | + | + |  |  |  |  |  | 5 | -99.246 | 208.7 | 3.55 | 0.061 |
| 56 | + | + | + |  | + | + |  | 8 | -96.165 | 208.9 | 3.74 | 0.055 |
| 32 | + | + | + | + | + |  |  | 8 | -96.185 | 209.0 | 3.78 | 0.054 |
| 8 | + | + | + |  |  |  |  | 6 | -98.867 | 210.1 | 4.89 | 0.031 |
| 12 | + | + |  | + |  |  |  | 6 | -99.224 | 210.8 | 5.61 | 0.022 |
| 64 | + | + | + | + | + | + |  | 9 | -96.115 | 211.0 | 5.79 | 0.020 |
| 40 | + | + | + |  |  | + |  | 7 | -98.652 | 211.8 | 6.58 | 0.013 |
| 16 | + | + | + | + |  |  |  | 7 | -98.835 | 212.1 | 6.95 | 0.011 |
| 128 | + | + | + | + | + | + | + | 10 | -96.069 | 213.1 | 7.87 | 0.007 |
| 48 | + | + | + | + |  | + |  | 8 | -98.614 | 213.8 | 8.64 | 0.005 |
| 1 |  |  |  |  |  |  |  | 3 | -108.352 | 222.8 | 17.62 | 0.000 |
| 5 |  |  | + |  |  |  |  | 4 | -107.836 | 223.8 | 18.65 | 0.000 |
| 3 |  | + |  |  |  |  |  | 4 | -108.352 | 224.9 | 19.68 | 0.000 |
| 7 |  | + | + |  |  |  |  | 5 | -107.831 | 225.9 | 20.72 | 0.000 |
| 39 |  | + | + |  |  | + |  | 6 | -107.440 | 227.2 | 22.04 | 0.000 |

ii) Recruit to fledglings ratio models with Δ AICc < 2

| Model | Residual Lay date | Age | Infection | Lay date* Age | Lay Date* Infection | Age* Infection | Lay date* Age* Infection | k | Log Likelihood | AICc | ΔAICc | Weight |
| --- | --- | --- | --- | --- | --- | --- | --- | --- | --- | --- | --- | --- |
| 22 | + |  | + |  | + |  |  | 6 | -96.420 | 205.2 | 0.00 | 0.55 |
| 24 | + | + | + |  | + |  |  | 7 | -96.216 | 206.9 | 1.71 | 0.24 |
| 2 | + |  |  |  |  |  |  | 4 | -99.471 | 207.1 | 1.92 | 0.21 |

iii) Recruits to fledglings ratio: relative variable importance based on e-ii)

| Residual Lay Date | Infection | Infection* Lay Date | Age |
| --- | --- | --- | --- |
| 1.00 | 0.79 | 0.79 | 0.24 |

1. Number of fledglings to clutch size ratio
2. All fledglings to clutch size ratio models

| Model | Residual Lay date | Age | Infection | Lay date* Age | Lay Date* Infection | Age* Infection | Lay date* Age* Infection | k | Log Likelihood | AICc | ΔAICc | Weight |
| --- | --- | --- | --- | --- | --- | --- | --- | --- | --- | --- | --- | --- |
| 1 |  |  |  |  |  |  |  | 3 | -178.400 | 362.9 | 0.00 | 0.243 |
| 3 |  | + |  |  |  |  |  | 4 | -177.621 | 363.4 | 0.51 | 0.189 |
| 5 |  |  | + |  |  |  |  | 4 | -178.355 | 364.9 | 1.97 | 0.091 |
| 2 | + |  |  |  |  |  |  | 4 | -178.371 | 364.9 | 2.01 | 0.089 |
| 4 | + | + |  |  |  |  |  | 5 | -177.515 | 365.3 | 2.38 | 0.074 |
| 7 |  | + | + |  |  |  |  | 5 | -177.612 | 365.5 | 2.57 | 0.067 |
| 39 |  | + | + |  |  | + |  | 6 | -177.088 | 366.5 | 3.62 | 0.040 |
| 12 | + | + |  | + |  |  |  | 6 | -177.229 | 366.8 | 3.90 | 0.035 |
| 6 | + |  | + |  |  |  |  | 5 | -178.321 | 366.9 | 3.99 | 0.033 |
| 22 | + |  | + |  | + |  |  | 6 | -177.383 | 367.1 | 4.21 | 0.030 |
| 8 | + | + | + |  |  |  |  | 6 | -177.504 | 367.4 | 4.45 | 0.026 |
| 24 | + | + | + |  | + |  |  | 7 | -176.584 | 367.6 | 4.73 | 0.023 |
| 40 | + | + | + |  |  | + |  | 7 | -176.996 | 368.5 | 5.55 | 0.015 |
| 16 | + | + | + | + |  |  |  | 7 | -177.215 | 368.9 | 5.99 | 0.012 |
| 56 | + | + | + |  | + | + |  | 8 | -176.249 | 369.1 | 6.19 | 0.011 |
| 32 | + | + | + | + | + |  |  | 8 | -176.355 | 369.3 | 6.41 | 0.010 |
| 48 | + | + | + | + |  | + |  | 8 | -176.741 | 370.1 | 7.18 | 0.007 |
| 64 | + | + | + | + | + | + |  | 9 | -176.024 | 370.8 | 7.90 | 0.005 |
| 128 | + | + | + | + | + | + | + | 10 | -176.024 | 373.0 | 10.07 | 0.002 |

1. Fledglings to clutch size ratio models with Δ AICc < 2

| Model | Residual Lay date | Age | Infection | Lay date* Age | Lay Date* Infection | Age* Infection | Lay date* Age* Infection | k | Log Likelihood | AICc | ΔAICc | Weight |
| --- | --- | --- | --- | --- | --- | --- | --- | --- | --- | --- | --- | --- |
| 1 |  |  |  |  |  |  |  | 3 | -178.400 | 362.9 | 0.00 | 0.47 |
| 3 |  | + |  |  |  |  |  | 4 | -177.621 | 363.4 | 0.51 | 0.36 |
| 5 |  |  | + |  |  |  |  | 4 | -178.355 | 364.9 | 1.97 | 0.17 |

iii) Fledglings to clutch size ratio: relative variable importance based on g-ii)

| Age | Infection |
| --- | --- |
| 0.36 | 0.17 |

g) Average Fledgling weight models

i) All average fledgling weight models

| Model | Residual Lay date | Age | Infection | Lay date* Age | Lay Date* Infection | Age* Infection | Lay date* Age* Infection | k | Log Likelihood | AICc | ΔAICc | Weight |
| --- | --- | --- | --- | --- | --- | --- | --- | --- | --- | --- | --- | --- |
| 1 |  |  |  |  |  |  |  | 4 | -181.040 | 370.4 | 0.00 | 0.501 |
| 5 |  |  | + |  |  |  |  | 5 | -180.341 | 371.1 | 0.76 | 0.343 |
| 3 |  | + |  |  |  |  |  | 5 | -181.914 | 374.3 | 3.90 | 0.071 |
| 7 |  | + | + |  |  |  |  | 6 | -181.211 | 375.1 | 4.69 | 0.048 |
| 39 |  | + | + |  |  | + |  | 7 | -181.217 | 377.3 | 6.93 | 0.016 |
| 2 | + |  |  |  |  |  |  | 5 | -183.990 | 378.4 | 8.06 | 0.009 |
| 6 | + |  | + |  |  |  |  | 6 | -183.352 | 379.4 | 8.97 | 0.006 |
| 16 | + | + | + | + |  |  |  | 8 | -182.056 | 381.3 | 10.87 | 0.002 |
| 12 | + | + |  | + |  |  |  | 7 | -183.360 | 381.6 | 11.21 | 0.002 |
| 4 | + | + |  |  |  |  |  | 6 | -184.817 | 382.3 | 11.90 | 0.001 |
| 8 | + | + | + |  |  |  |  | 7 | -184.172 | 383.2 | 12.84 | 0.001 |
| 48 | + | + | + | + |  | + |  | 9 | -181.945 | 383.3 | 12.94 | 0.001 |
| 22 | + |  | + |  | + |  |  | 7 | -185.235 | 385.4 | 14.96 | 0.000 |
| 40 | + | + | + |  |  | + |  | 8 | -184.195 | 385.5 | 15.14 | 0.000 |
| 32 | + | + | + | + | + |  |  | 9 | -184.052 | 387.5 | 17.16 | 0.000 |
| 24 | + | + | + |  | + |  |  | 8 | -186.065 | 389.3 | 18.89 | 0.000 |
| 64 | + | + | + | + | + | + |  | 10 | -183.781 | 389.3 | 18.95 | 0.000 |
| 56 | + | + | + |  | + | + |  | 9 | -185.972 | 391.4 | 21.00 | 0.000 |
| 128 | + | + | + | + | + | + | + | 11 | -183.841 | 391.8 | 21.44 | 0.000 |

ii) Average fledgling weight models with Δ AICc < 2

| Model | Residual Lay date | Age | Infection | Lay date* Age | Lay Date* Infection | Age* Infection | Lay date* Age* Infection | k | Log Likelihood | AICc | ΔAICc | Weight |
| --- | --- | --- | --- | --- | --- | --- | --- | --- | --- | --- | --- | --- |
| 1 |  |  |  |  |  |  |  | 4 | -181.040 | 370.4 | 0.00 | 0.59 |
| 5 |  |  | + |  |  |  |  | 5 | -180.341 | 371.1 | 0.76 | 0.41 |

iii) Average fledgling weight: relative variable importance based on g-ii)

| Infection |
| --- |
| 0.41 |
