# Supplementary material for: Malaria-Infected Female Collared Flycatchers (Ficedula albicollis) Do Not Pay the Cost of Late Breeding
Source: PLoS One. 2014 Jan 23;9(1):e85822. doi: 10.1371/journal.pone.0085822 (PMC3900437; doi:10.1371/journal.pone.0085822)
Supplement: Table S4 — Primer sequences used for real-time qPCR. Genus-specific primers amplify a fragment of parasite's cytochrome b gene; host primers amplify an ultra-conserved of the host nuclear DNA. (DOC) [file pone.0085822.s004.doc]

| Target | Primers | Sequences (5’-3’) | Size (bp) |
| --- | --- | --- | --- |
| *Haemoproteus* | FihaF | 5’-GGATATATTATTAGTGATCCAACT-3’ | 108 |
|  | FihaR | 5'- GACATAATAGACTTGGATAGAAA -3' |  |
|  |  |  |  |
| Host primers | SFSR3Fb | 5’-ACTAGCCCTTTCAGCGTCATGT-3’ | 114 |
|  | SFSR3Rb | 5’-CATGCTGGGGAACCAAAGG- 3’ |  |

Appendix Table S4. Primer sequences used for real-time qPCR. Genus-specific primers amplify a fragment of parasite’s cytochrome b gene; host primers amplify an ultra-conserved of the host nuclear DNA
