# Supplementary material for: Malaria-Infected Female Collared Flycatchers (Ficedula albicollis) Do Not Pay the Cost of Late Breeding
Source: PLoS One. 2014 Jan 23;9(1):e85822. doi: 10.1371/journal.pone.0085822 (PMC3900437; doi:10.1371/journal.pone.0085822)
Supplement: Table S5 — Reproductive success and infection intensity model selection tables for a subset (see Materials and Methods section for details) individuals infected with hPHSIB1 lineage or uninfected females. (DOC) [file pone.0085822.s005.doc]

**Appendix S5**. Reproductive success and infection intensity model selection tables

1. Lay date

i) All lay date models

| Model | Age | Infection intensity | Age * Infection intensity | k | Log Likelihood | AICc | Δ AICc | Weight |
| --- | --- | --- | --- | --- | --- | --- | --- | --- |
| 8 | + | + | + | 7 | -307.073 | 629.3 | 0.00 | 0.461 |
| 2 | + |  |  | 5 | -309.930 | 630.4 | 1.19 | 0.254 |
| 4 | + | + |  | 6 | -309.471 | 631.8 | 2.51 | 0.131 |
| 1 |  |  |  | 4 | -311.991 | 632.4 | 3.11 | 0.097 |
| 3 |  | + |  | 5 | -311.437 | 633.5 | 4.20 | 0.056 |

ii) Lay date models with Δ AICc < 2

| Model | Age | Infection intensity | Age * Infection intensity | k | Log Likelihood | AICc | Δ AICc | Weight |
| --- | --- | --- | --- | --- | --- | --- | --- | --- |
| 8 | + | + | + | 7 | -307.073 | 629.3 | 0.00 | 0.64 |
| 2 | + |  |  | 5 | -309.930 | 630.4 | 1.19 | 0.36 |

iii) Lay date: relative variable importance based on a-ii)

| Age | Infection intensity | Age * Infection intensity |
| --- | --- | --- |
| 1.0 | 0.64 | 0.64 |

iv) Lay date: averaged model estimates

| **Parameter** | **Estimate** | **SE** | **Lower 95% CI** | **Upper 95% CI** |
| --- | --- | --- | --- | --- |
| Age (yearling) | 1.1468 | 0.9920 | -0.7974425 | 3.0909653 |
| Infection intensity | -0.4055 | 0.5596 | -1.5023173 | 0.6913722 |
| Age * intensity | 2.6875 | 2.8372 | -2.8733264 | 8.2483229 |

1. Clutch size

i) All clutch size models

| Model | Residual Lay date | Age | Infection intensity | Lay date* Age | Lay Date* Infection intensity | Age* Infection intensity | Lay date* Age* Infection intensity | k | Log Likelihood | AICc | Δ AICc | Weight |
| --- | --- | --- | --- | --- | --- | --- | --- | --- | --- | --- | --- | --- |
| 1 |  |  |  |  |  |  |  | 3 | -8.228 | 22.7 | 0.00 | 0.338 |
| 2 | + |  |  |  |  |  |  | 4 | -7.932 | 24.2 | 1.56 | 0.155 |
| 3 |  | + |  |  |  |  |  | 4 | -8.049 | 24.5 | 1.80 | 0.138 |
| 5 |  |  | + |  |  |  |  | 4 | -8.226 | 24.8 | 2.15 | 0.115 |
| 4 | + | + |  |  |  |  |  | 5 | -7.799 | 26.2 | 3.50 | 0.059 |
| 6 | + |  | + |  |  |  |  | 5 | -7.932 | 26.4 | 3.76 | 0.052 |
| 7 |  | + | + |  |  |  |  | 5 | -8.049 | 26.7 | 4.00 | 0.046 |
| 12 | + | + |  | + |  |  |  | 6 | -7.793 | 28.4 | 5.73 | 0.019 |
| 8 | + | + | + |  | + |  |  | 6 | -7.798 | 28.4 | 5.73 | 0.019 |
| 2 | + |  | + |  |  |  |  | 6 | -7.915 | 28.7 | 5.97 | 0.017 |
| 39 |  | + | + |  |  | + |  | 6 | -7.946 | 28.7 | 6.03 | 0.017 |
| 40 | + | + | + |  |  | + |  | 7 | -7.676 | 30.5 | 7.78 | 0.007 |
| 24 | + | + | + |  | + |  |  | 7 | -7.780 | 30.7 | 7.98 | 0.006 |
| 16 | + | + | + | + |  |  |  | 7 | -7.793 | 30.7 | 8.01 | 0.006 |
| 48 | + | + | + | + |  | + |  | 8 | -7.667 | 32.8 | 10.09 | 0.002 |
| 56 | + | + | + |  | + | + |  | 8 | -7.671 | 32.8 | 10.10 | 0.002 |
| 32 | + | + | + | + | + |  |  | 8 | -7.777 | 33.0 | 10.31 | 0.002 |
| 64 | + | + | + | + | + | + |  | 9 | -7.664 | 35.1 | 12.46 | 0.001 |
| 128 | + | + | + | + | + | + | + | 10 | -7.652 | 37.5 | 14.86 | 0.000 |

ii) Clutch size models with Δ AICc < 2

| Model | Residual Lay date | Age | Infection intensity | Lay date* Age | Lay Date* Infection intensity | Age* Infection intensity | Lay date* Age* Infection intensity | k | Log Likelihood | AICc | Δ AICc | Weight |
| --- | --- | --- | --- | --- | --- | --- | --- | --- | --- | --- | --- | --- |
| 1 |  |  |  |  |  |  |  | 3 | -8.228 | 22.7 | 0.00 | 0.54 |
| 2 | + |  |  |  |  |  |  | 4 | -7.932 | 24.2 | 1.56 | 0.25 |
| 3 |  | + |  |  |  |  |  | 4 | -8.049 | 24.5 | 1.80 | 0.22 |

iii) Clutch size: relative variable importance based on b-ii)

| Residual Lay Date | Age |
| --- | --- |
| 0.25 | 0.22 |

1. Number of fledglings models
2. All number of fledglings models

| Model | Residual Lay date | Age | Infection intensity | Lay date* Age | Lay Date* Infection intensity | Age* Infection intensity | Lay date* Age* Infection intensity | k | Log Likelihood | AICc | Δ AICc | Weight |
| --- | --- | --- | --- | --- | --- | --- | --- | --- | --- | --- | --- | --- |
| 1 |  |  |  |  |  |  |  | 3 | -40.558 | 87.4 | 0.00 | 0.252 |
| 3 |  | + |  |  |  |  |  | 4 | -39.521 | 87.5 | 0.11 | 0.238 |
| 5 |  |  | + |  |  |  |  | 4 | -40.446 | 89.3 | 1.96 | 0.095 |
| 2 | + |  |  |  |  |  |  | 4 | -40.558 | 89.6 | 2.18 | 0.085 |
| 7 |  | + | + |  |  |  |  | 5 | -39.475 | 89.6 | 2.24 | 0.082 |
| 4 | + | + |  |  |  |  |  | 5 | -39.493 | 89.7 | 2.28 | 0.080 |
| 12 | + | + |  | + |  |  |  | 6 | -39.285 | 91.5 | 4.14 | 0.032 |
| 6 | + |  | + |  |  |  |  | 5 | -40.445 | 91.6 | 4.19 | 0.031 |
| 39 |  | + | + |  |  | + |  | 6 | -39.366 | 91.7 | 4.31 | 0.029 |
| 8 | + | + | + |  |  |  |  | 6 | -39.443 | 91.8 | 4.46 | 0.027 |
| 22 | + |  | + |  | + |  |  | 6 | -40.409 | 93.8 | 6.39 | 0.010 |
| 16 | + | + | + | + |  |  |  | 7 | -39.244 | 93.8 | 6.40 | 0.010 |
| 40 | + | + | + |  |  | + |  | 7 | -39.330 | 93.9 | 6.57 | 0.009 |
| 24 | + | + | + |  | + |  |  | 7 | -39.394 | 94.1 | 6.69 | 0.009 |
| 48 | + | + | + | + |  | + |  | 8 | -39.120 | 95.9 | 8.53 | 0.004 |
| 32 | + | + | + | + | + |  |  | 8 | -39.228 | 96.1 | 8.75 | 0.003 |
| 56 | + | + | + |  | + | + |  | 8 | -39.301 | 96.3 | 8.90 | 0.003 |
| 64 | + | + | + | + | + | + |  | 9 | -39.115 | 98.3 | 10.97 | 0.001 |
| 128 | + | + | + | + | + | + | + | 10 | -39.115 | 100.8 | 13.47 | 0.000 |

ii) Number of fledglings models with Δ AICc < 2

| Model | Residual Lay date | Age | Infection intensity | Lay date* Age | Lay Date* Infection intensity | Age* Infection intensity | Lay date* Age* Infection intensity | k | Log Likelihood | AICc | Δ AICc | Weight |
| --- | --- | --- | --- | --- | --- | --- | --- | --- | --- | --- | --- | --- |
| 1 |  |  |  |  |  |  |  | 3 | -40.558 | 87.4 | 0.00 | 0.43 |
| 3 |  | + |  |  |  |  |  | 4 | -39.521 | 87.5 | 0.11 | 0.41 |
| 5 |  |  | + |  |  |  |  | 4 | -40.446 | 89.3 | 1.96 | 0.16 |

1. Number of fledglings: relative variable importance based on g-ii)

| Age | Infection intensity |
| --- | --- |
| 0.41 | 0.16 |

d) Number of recruits

i) All number of recruits models

| Model | Residual Lay date | Age | Infection intensity | Lay date* Age | Lay Date* Infection intensity | Age* Infection intensity | Lay date* Age* Infection intensity | k | Log Likelihood | AICc | Δ AICc | Weight |
| --- | --- | --- | --- | --- | --- | --- | --- | --- | --- | --- | --- | --- |
| 1 |  |  |  |  |  |  |  | 3 | -40.602 | 87.5 | 0.00 | 0.371 |
| 5 |  |  | + |  |  |  |  | 4 | -40.465 | 89.4 | 1.91 | 0.143 |
| 3 |  | + |  |  |  |  |  | 4 | -40.549 | 89.5 | 2.08 | 0.131 |
| 2 | + |  |  |  |  |  |  | 4 | -40.601 | 89.6 | 2.18 | 0.125 |
| 7 |  | + | + |  |  |  |  | 5 | -40.346 | 91.4 | 3.90 | 0.053 |
| 6 | + |  | + |  |  |  |  | 5 | -40.461 | 91.6 | 4.13 | 0.047 |
| 4 | + | + |  |  |  |  |  | 5 | -40.549 | 91.8 | 4.30 | 0.043 |
| 39 |  | + | + |  |  | + |  | 6 | -40.283 | 93.5 | 6.05 | 0.01 |
| 8 | + | + | + |  |  |  |  | 6 | -40.345 | 93.6 | 6.18 | 0.017 |
| 22 | + |  | + |  | + |  |  | 6 | -40.455 | 93.9 | 6.40 | 0.015 |
| 12 | + | + |  | + |  |  |  | 6 | -40.473 | 93.9 | 6.43 | 0.015 |
| 16 | + | + | + | + |  |  |  | 7 | -40.243 | 95.8 | 8.31 | 0.006 |
| 40 | + | + | + |  |  | + |  | 7 | -40.283 | 95.9 | 8.38 | 0.006 |
| 24 | + | + | + |  | + |  |  | 7 | -40.341 | 96.0 | 8.50 | 0.005 |
| 48 | + | + | + | + |  | + |  | 8 | -40.167 | 98.0 | 10.54 | 0.002 |
| 32 | + | + | + | + | + |  |  | 8 | -40.243 | 98.2 | 10.69 | 0.002 |
| 56 | + | + | + |  | + | + |  | 8 | -40.283 | 98.2 | 10.77 | 0.002 |
| 64 | + | + | + | + | + | + |  | 9 | -40.158 | 100.4 | 12.96 | 0.001 |
| 128 | + | + | + | + | + | + | + | 10 | -39.935 | 102.5 | 15.02 | 0.000 |

ii) Number of recruit models with Δ AICc < 2

| Model | Residual Lay date | Age | Infection intensity | Lay date* Age | Lay Date* Infection intensity | Age* Infection intensity | Lay date* Age* Infection intensity | k | Log Likelihood | AICc | Δ AICc | Weight |
| --- | --- | --- | --- | --- | --- | --- | --- | --- | --- | --- | --- | --- |
| 1 |  |  |  |  |  |  |  | 3 | -40.602 | 87.5 | 0.00 | 0.72 |
| 5 |  |  | + |  |  |  |  | 4 | -40.465 | 89.4 | 1.91 | 0.28 |

iii) Recruit relative variable importance based on d-ii)

| Infection intensity |
| --- |
| 0.28 |

1. Average fledgling weight models
2. All average fledgling weight models

| Model | Residual Lay date | Age | Infection intensity | Lay date* Age | Lay Date* Infection intensity | Age* Infection intensity | Lay date* Age* Infection intensity | k | Log Likelihood | AICc | Δ AICc | Weight |
| --- | --- | --- | --- | --- | --- | --- | --- | --- | --- | --- | --- | --- |
| 1 |  |  |  |  |  |  |  | 4 | -64.256 | 137.3 | 0.00 | 0.454 |
| 3 |  | + |  |  |  |  |  | 5 | -63.841 | 138.8 | 1.56 | 0.208 |
| 39 |  | + | + |  |  | + |  | 7 | -61.941 | 140.1 | 2.82 | 0.111 |
| 5 |  |  | + |  |  |  |  | 5 | -65.006 | 141.1 | 3.89 | 0.065 |
| 7 |  | + | + |  |  |  |  | 6 | -64.002 | 141.6 | 4.37 | 0.051 |
| 2 | + |  |  |  |  |  |  | 5 | -65.290 | 141.7 | 4.46 | 0.049 |
| 4 | + | + |  |  |  |  |  | 6 | -64.382 | 142.4 | 5.13 | 0.035 |
| 40 | + | + | + |  |  | + |  | 8 | -62.764 | 144.4 | 7.15 | 0.013 |
| 8 | + | + | + |  |  |  |  | 7 | -64.916 | 146.0 | 8.78 | 0.006 |
| 6 | + |  | + |  |  |  |  | 6 | -66.356 | 146.3 | 9.08 | 0.005 |
| 12 | + | + |  | + |  |  |  | 7 | -66.557 | 149.3 | 12.06 | 0.001 |
| 56 | + | + | + |  | + | + |  | 9 | -64.069 | 149.8 | 12.56 | 0.001 |
| 22 | + |  | + |  | + |  |  | 7 | -67.007 | 150.2 | 12.96 | 0.001 |
| 24 | + | + | + |  | + |  |  | 8 | -66.179 | 151.2 | 13.99 | 0.000 |
| 48 | + | + | + | + |  | + |  | 9 | -64.992 | 151.7 | 14.40 | 0.000 |
| 16 | + | + | + | + |  |  |  | 7 | -67.138 | 153.2 | 15.90 | 0.000 |
| 64 | + | + | + | + | + | + |  | 10 | -66.302 | 157.2 | 19.94 | 0.000 |
| 128 | + | + | + | + | + | + | + | 11 | -65.177 | 158.0 | 20.72 | 0.000 |
| 32 | + | + | + | + | + |  |  | 9 | -68.443 | 158.6 | 21.31 | 0.000 |

ii) Average fledgling weight models with ΔAICc < 2

| Model | Residual Lay date | Age | Infection intensity | Lay date* Age | Lay Date* Infection intensity | Age* Infection intensity | Lay date* Age* Infection intensity | k | Log Likelihood | AICc | Δ AICc | Weight |
| --- | --- | --- | --- | --- | --- | --- | --- | --- | --- | --- | --- | --- |
| 1 |  |  |  |  |  |  |  | 4 | -64.256 | 137.3 | 0.00 | 0.69 |
| 3 |  | + |  |  |  |  |  | 5 | -63.841 | 138.8 | 1.56 | 0.31 |
